# Functional Diversity and Structural Disorder in the Human Ubiquitination Pathway

**DOI:** 10.1371/journal.pone.0065443

**Published:** 2013-05-29

**Authors:** Pallab Bhowmick, Rita Pancsa, Mainak Guharoy, Peter Tompa

**Affiliations:** 1 VIB Department of Structural Biology, Vrije Universiteit Brussel, Brussels, Belgium; 2 Institute of Enzymology, Research Centre for Natural Sciences, Hungarian Academy of Sciences, Budapest, Hungary; Ecole Polytechnique Federale de Lausanne, Switzerland

## Abstract

The ubiquitin-proteasome system plays a central role in cellular regulation and protein quality control (PQC). The system is built as a pyramid of increasing complexity, with two E1 (ubiquitin activating), few dozen E2 (ubiquitin conjugating) and several hundred E3 (ubiquitin ligase) enzymes. By collecting and analyzing E3 sequences from the KEGG BRITE database and literature, we assembled a coherent dataset of 563 human E3s and analyzed their various physical features. We found an increase in structural disorder of the system with multiple disorder predictors (IUPred – E1: 5.97%, E2: 17.74%, E3: 20.03%). E3s that can bind E2 and substrate simultaneously (single subunit E3, ssE3) have significantly higher disorder (22.98%) than E3s in which E2 binding (multi RING-finger, mRF, 0.62%), scaffolding (6.01%) and substrate binding (adaptor/substrate recognition subunits, 17.33%) functions are separated. In ssE3s, the disorder was localized in the substrate/adaptor binding domains, whereas the E2-binding RING/HECT-domains were structured. To demonstrate the involvement of disorder in E3 function, we applied normal modes and molecular dynamics analyses to show how a disordered and highly flexible linker in human CBL (an E3 that acts as a regulator of several tyrosine kinase-mediated signalling pathways) facilitates long-range conformational changes bringing substrate and E2-binding domains towards each other and thus assisting in ubiquitin transfer. E3s with multiple interaction partners (as evidenced by data in STRING) also possess elevated levels of disorder (hubs, 22.90% vs. non-hubs, 18.36%). Furthermore, a search in PDB uncovered 21 distinct human E3 interactions, in 7 of which the disordered region of E3s undergoes induced folding (or mutual induced folding) in the presence of the partner. In conclusion, our data highlights the primary role of structural disorder in the functions of E3 ligases that manifests itself in the substrate/adaptor binding functions as well as the mechanism of ubiquitin transfer by long-range conformational transitions.

## Introduction

Proper functioning of a eukaryotic cell rests on a fine balance between the synthesis and degradation of the thousands of its proteins, i.e. proteostasis [1]. A major guardian of proteostasis is the protein quality control (PQC) system, which ensures folding of proteins to their native structure and their degradation if they become superfluous or irreparably misfolded. Folding is assisted by molecular chaperones [2], whereas degradation is orchestrated by the ubiquitin-proteasome system (UPS), which tags misfolded proteins or proteins, the action of which needs to be terminated, with a covalently attached polyubiquitin chain for degradation by the 26S proteasome. Ubiquitination also has degradation-independent regulatory roles, because the attachment of a single ubiquitin moiety (mono-ubiquitination), multiple ubiquitin moieties (multi-ubiquitination) and even polyubiquitination through different chain topologies (linking through Lysine63, for example), modulate, rather than terminate, the action of proteins in diverse cellular processes, such as transcription, endocytosis and cell-death [3], [4].

Ubiquitin is an extremely conserved protein of 76 amino acids, usually attached to a Lysine residue of the target protein via its C-terminal carboxyl group through an isopeptide bond. Ubiquitin itself has seven Lys residues, which enable complex chain extensions with distinct functional outcomes. Attachment of ubiquitin is carried out by a series of proteins having ubiquitin activating (E1), ubiquitin conjugating (E2) and ubiquitin ligase (E3) activities. Structural and functional interplay of these enzymes and their accessory proteins is crucial in controlling the activation and transfer of ubiquitin to target proteins [5]. The system of ubiquitination is built as a pyramid, reflecting the increasing functional complexity leading from ubiquitin to the degradome/ubiquitinome within the proteome.

Two E1s identified in the human genome are responsible for the chemical activation of ubiquitin. So far, more than 30 E2s have been identified: most of them contain a highly conserved ubiquitin conjugation (UBC) domain [4], [6] that forms a covalent intermediate with ubiquitin via its catalytic Cys residue. Most diverse is the family of E3 proteins, which bring together ubiquitin-charged E2 (E2∼Ub) and the substrate protein; they bind to their substrate either directly or through adaptor/substrate recognition proteins [7]–[9]. The E3 family is commensurable in functional complexity with the kinome [10]: based on functional and sequence criteria, 617 E3s have been suggested to exist in the human genome [11]. E3s are classified into two basic types: HECT (homologous to E6-AP carboxyl terminus) E3s form an intermediate thioester bond with ubiquitin [7], whereas RING (really interesting new gene), and the related U-box E3s do not [8], [9]; rather they bind both to E2∼Ub and the substrate to assist the transfer of the ubiquitin moiety. In single-subunit E3s (ssE3s) such as single RING-finger (sRF), U-box and HECT E3s, the E3 binds E2∼Ub and the substrate simultaneously, and requires no accessory protein for action (although they are not necessarily monomeric in their active state). On the contrary, multi-subunit E3s (msE3s, also termed cullin-RING ligases, CRLs) form complexes in which E2∼Ub binding by multi RING-finger (mRF) and substrate binding by adaptor/substrate recognition (such as APC (anaphase promoting complex), ADAP (adaptor), VHL (Von Hippel-Lindau disease tumor suppressor), DCAF (DDB1 and CUL4 associated factor), BTB (Broad Complex/Tramtrack/Bric-a-Brac), F-box and SOCS (suppressor of cytokine signaling)) subunits are separated and connected by scaffolding cullin (CUL) proteins. The best studied msE3 complex is SCF (Skp1-cullin-F-box), which belongs to the CRL family [9], [12].

Overall, the UPS system is a very complicated and highly regulated network of proteins that can distinguish between folded and misfolded states of its substrates far exceeding its components in number. To address and possibly resolve the underlying contradiction between coverage of the entire ubiquitinated complement of the proteome (degradome, ubiquitinome) and specificity for certain cellular situations and/or structural states, we decided to look for intrinsic structural disorder in the E3 proteins of the ubiquitin system and decipher the role(s) that intrinsic protein flexibility and disorder may play in the ubiquitination pathway. Many proteins or regions of proteins (intrinsically disordered proteins/regions, IDPs/IDRs) exist and function without a well-defined structural state, which provides distinct functional advantages [13]–[15]. Structural disorder is increased in proteins playing signalling and/or regulatory roles, in which it may either enable flexible connection between binding elements (entropic chain function) or is directly involved in molecular recognition, harbouring short binding motifs or domains [16], [17]. In these functions, structural disorder provides many advantages, such as separation of specificity and binding strength, increased speed of interaction, adaptability in binding, binding promiscuity/moonlighting [18], and regulation by post-translational modifications [13]–[15]; these are all relevant for the functional challenges of the UPS system. A few isolated observations have previously shown evidence of structural disorder in members of the ubiquitination pathway (e.g. MDM2 [19] and BRCA1 [20]), and pinpointed the direct involvement of structural disorder of the substrate (e.g. Sic1 [21]) or the E3 itself (e.g. San1 [22]) in substrate recognition. In this work we establish a near-exhaustive database of human components of the E1-E2-E3 system obtained from both the KEGG BRITE server and perusal of the literature, and show - by systematic bioinformatics analysis - an elevated level and extended use of disordered regions/domains in this system. We show that structural disorder of E3s is often involved in substrate/partner recognition, and we also include a detailed case study on the human E3 ligase c-CBL to demonstrate the mechanism by which highly flexible regions (that are also predicted to be disordered) facilitate transfer of the ubiquitin moiety.

## Results

### Dataset of the human ubiquitination system

**Figure 1:**
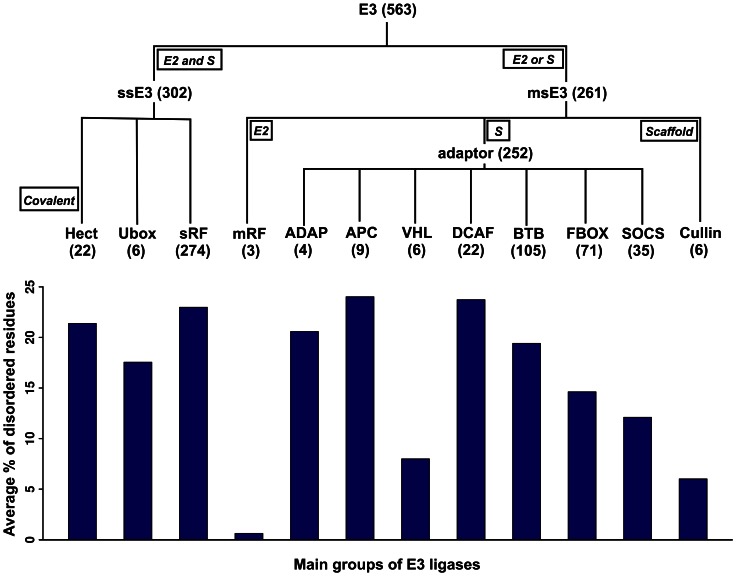
Predicted disorder of the main classes of human E3 ubiquitin ligases. We used the IUPred disorder prediction method for predicting structural disorder in 563 human E3 ligases, and calculated the average percent of disordered residues for proteins in the different sub-classes. The functional classification tree for the E3 family is shown above the bars. The specific functional characteristics for each main branch are indicated in boxes, such as interaction with E2 enzyme and/or with the substrate (‘S’), transient covalent binding to ubiquitin (‘covalent’), or functioning as a scaffold or adaptor/substrate recognition subunit in msE3s.

We assembled a comprehensive dataset of proteins involved in the human ubiquitination system from data available in the literature [11] and in the KEGG BRITE database [23]–[25]. Our carefully curated database contains 2 E1, 29 E2 and 563 E3 enzymes (Tables S1, S2, S3 and Table 1); according to our knowledge this is the first comprehensive, manually curated collection of all experimentally validated members of the human ubiquitination system. The workflow of data collection, filtering and merging is detailed in Methods (see also Figure S1). We adopted the classification of E3 proteins (Table 1, Figure 1) from the literature [11].

**Table 1:** Overall disorder content of ubiquitin associated enzymes.

Enzymes	Average length	No. of proteins	Average% of disordered residues	Average no. of disordered residues	No. of mostly disordered E3s (>50%)	No. of E3s with long (> = 30) disordered regions
E1	1055	2	5.97	63	0	1
E2	413	29	17.74	81	0	13
Different human E3 ubiquitin ligase families						
Ubox	706	6	17.55	124	0	4
Hect	1720	22	21.38	401	0	17
sRF	640	274	22.98	159	31	164
mRF^a^	102	3	0.62	1	0	0
*Total*	*714*	*305*	*22.54*	*175*	*31*	*185*
ADAP	383	4	20.58	30	0	0
APC	677	9	24.02	83	2	6
CUL^a^	790	6	6.01	49	0	1
VHL^a^	587	6	7.99	22	0	1
DCAF	792	22	23.73	232	1	16
BTB	624	105	19.41	133	10	60
F-box	563	71	14.62	101	3	33
SOCS^a^	389	35	12.10	88	1	11
*Total*	*591*	*258*	*17.07*	*114*	*17*	*128*
*All E3s*	*657*	*563*	*20.03*	*146*	*48*	*313*

sRF, HECT and U-box families belong to the single subunit subclass, mRF is the only family of the multi subunit subclass, and all the other families are E3 adaptor proteins.

a These categories of E3 proteins have significantly lower average% of disordered residues as compared to the E2 family. Mann-Whitney U-tests were used for the statistical significance testing.

E3 proteins are able to transfer ubiquitin directly to their target protein by binding E2∼Ub and substrate simultaneously (ssE3s: HECT, RING-finger and U-box); others assemble into complexes (msE3s), in which E2∼Ub bound by a multi RING-finger protein (mRFs: ANAPC11, RBX1, and RNF7) is connected to adaptor/substrate recognition subunits (ADAP, APC, VHL, DCAF, BTB, F-box and SOCS) via a scaffold protein cullin (CUL). Our final dataset contained 302 ssE3s and 261 msE3s.

### Disorder content of E3 ubiquitin ligases

First, we predicted intrinsic protein disorder with IUPred for the E1, E2 and E3 proteins in our database and compared their overall disorder content (Table 1). We observed an increase in predicted disorder with E1s having the lowest, and E3s having the highest levels (E1<E2<E3). The statistics were re-calculated using two other disorder predictors (VSL2 [26] and FoldIndex [27]) and the trend remained unchanged (Table S4). E3s were the most disordered in all the distinct measures we calculated (i.e., average number and percentage of disordered residues, number of mostly disordered proteins and the number of proteins with long disordered regions). The difference between the families is most conspicuous in the ratio of mostly disordered proteins (those with greater than 50% predicted disordered residues): there is no such protein among E1 and E2 classes, whereas there are 48 in E3s. This distribution reflects the increasing complexity in ubiquitination, also apparent in the number of proteins involved (E1: 2, E2: 29, E3: 563) and the number of their interacting partners. Given that the mean length of E3s is shorter than E1s, the increased disorder seen in E3s is even more significant in terms of their mean number of disordered residues (E1: 63.0, E2: 81.4, E3: 146.7; Mann-Whitney U-test (see Methods) p-value E2 vs E3: 0.016).

To unveil specific function-related properties we compared disorder between different E3 subcategories (Table 1). In the single-subunit subclass, RING (sRF) and HECT proteins have higher mean disorder content than U-box proteins. About 60% of sRF and 77% of HECT proteins have at least one long (≥30 residues) disordered region, and, given their extreme length (1720 residues), HECT proteins excel in the total number of disordered residues. In fact, HECTs are huge proteins consisting of many long disordered segments intermixed with ordered domains [7]. The overall picture that emerges is that ssE3s constitute a rather homogeneous class of long and significantly disordered (with a mean around 22%) proteins.

On the contrary, msE3s (mRFs and the accessory proteins) are extremely varied both in terms of length and disorder, reflecting their functional specialization. mRF E3s are the shortest, consisting of a single RING-finger domain and they are also the least disordered among the E3 families (<1% predicted disorder). Second to mRFs are the cullins (CUL: 6.0%), in accord with their role as rigid scaffolds in the assembly of msE3 complexes. In contrast, the proteins involved in substrate binding (adaptors and substrate recognition subunits) have disorder levels approaching that of ssE3s (8–25%). This observed difference in the disorder levels of substrate binding vs. scaffolding regions is also clearly noticeable within the ssE3 and mRF families, where the two different functionalities (E2-binding and substrate/adaptor binding) are combined within the same polypeptide chain. Using domain definitions from UniProt [28] and the disorder scores from the whole-protein predictions, we calculated the average disorder separately for the E2 binding domains (RING/HECT/U-box) of ssE3s and for the remaining (non-E2-binding) regions (where the substrate/adaptor binding functions are localized), discarding only transmembrane segments from the latter (Table 2). In all families (HECT, sRF, mRF) except for the U-box, the E2 binding regions are almost entirely structured (avg. disorder 1%), whereas the disorder is concentrated in the non-E2 binding regions (24.6%) (p<2.2E-16). The apparently contradictory result for the U-box sub-family may be due to the paucity of data (6 members) or may reflect an underlying functional difference with the other ssE3 sub-families. A possible explanation could be the fact that the U-box domain, unlike classical RING-domains, does not contain the hallmark zinc-coordinating residues that stabilize the cross-brace structure of the RING. The U-box scaffold is stabilized by salt bridge and hydrogen bonding interactions mediated by strongly conserved charged and polar residues [29]. The significantly higher disorder predicted for the U-box (compared to the RING-domains) may stem from the fact that classical predictors often assign higher disorder values to charged and polar residues.

**Table 2:** Disorder for E2-binding and non-E2-binding regions in E3 ligases.

E3 Family	Avg disorder content E2-binding domain^a^ (%)	Avg disorder content non-E2-binding regions^b^ (%)	P-value^c^
Ubox	18.9	18.2	0.5314
Hect	1.2	28.8	1.509e-07
SRF	0.8	24.7	<2.2e-16
MRF	0.0	1.1	0.2525
**Total**	**1.3**	**24.6**	**<2.2e-16**

a E2-binding domains include RING/U-box/HECT domains as taken from UniProt.

b All regions excluding RING/U-box/HECT domains and transmembrane segments based on UniProt.

c P-values calculated using the one-tailed Mann-Whitney U-test corresponding to the hypothesis that non-E2-binding domains are significantly more disordered than E2-binding domains.

**Figure 2:**
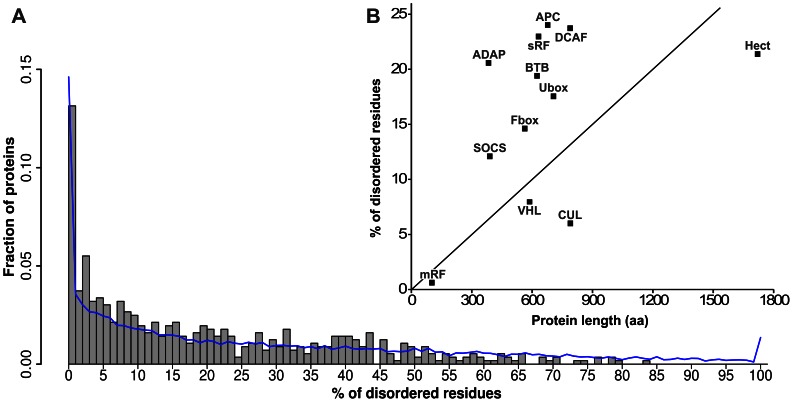
Distribution of the disorder content of E3s. Residue-level structural disorder was predicted for all 563 human E3 ligases by IUPred, and the percent of disordered residues was calculated for each protein. **A**) The distribution of E3 proteins as a function of their disorder content. The superposed line shows the disorder tendency for the human proteome (30% sequence redundancy). **B**) The average percent of disordered residues as a function of the mean sequence length for different E3 families.

For a large majority of E3s (464/563) the ratio of disordered residues is between 0% and 40% (Figure 2A) but there are also several (48) which are mostly disordered (>50%). The majority of the latter are found among ssE3s and in the BTB family of adaptors, both of which actually combine adaptor and substrate recognition functions in a single polypeptide chain [9], [12]. At the other extreme, for 74 E3 proteins (sRF: 26, HECT: 1, msRF: 2, CUL: 1, ADAP: 1, BTB: 15, DCAF: 1, F-box: 18, SOCS: 6 and VHL: 3) the ratio of disordered residues is close to 0. Thus, the distribution of structural disorder of E3s shows an excess of proteins with very low (0–5%) and also very high (40–100%) disorder, which is reminiscent of the power-law (scale-free) distribution of disorder in the entire proteome [30]. Such a distribution most likely indicates the functional importance of this feature. Figure 2A also includes a comparison with the distribution of predicted disorder for the human proteome (14180 sequences at 30% sequence identity). However, we did not find any statistically significant difference between the observed disorder distribution for the E3 sample and the human proteome (p>0.01). Since in our study we focus on the role of disordered regions, we repeated the analyses in Figure 2A considering only the fraction of protein residues occurring in long disordered regions (LDRs). The results are shown in Figure S2A. For 147/563 and 35/563 E3s, respectively, more than 25 and 50% of their residues are located within LDRs. We also focussed on the abundance of LDRs (defined as contiguous stretches of 30 or more predicted disordered residues; intervening sections of 3 or less ‘ordered’ residues were ignored). Figure S2B shows the distribution of LDRs in the E3 proteins and, for comparison, in the human proteome set. More than 50% of E3s possess at least one LDR emphasizing the functional importance of disorder in E3s; however, E3s are not significantly different in terms of LDR occurrence compared to the human proteome (p>0.01).

Although the E3 family does not differ markedly in overall terms compared with the human proteome (Figures 2A and S2); nonetheless, quantitatively similar disorder in different protein families might manifest itself in strikingly different functional and mechanistic terms. Therefore, in this manuscript we illustrate and characterize the manner in which structural disorder manifests itself in ubiquitination pathways, and elucidate the specific mechanisms by which E3 enzymes use structural disorder. The importance of disorder is probably also manifested in its broad correlation with protein length (Figure 2B), which suggests a disproportionately large amount of disordered residues (regions) in longer proteins. This underlying adaptive evolutionary drive is probably also underscored by the outliers: the rigid scaffold cullins [9], [12], which have less, and sRF E3s, or adaptor/substrate recognition subunits of msE3s (in particular DCAF, APC, and ADAP), which have more disorder than expected by their length (cf. Figure 1).

### Interaction Classification

**Figure 3:**
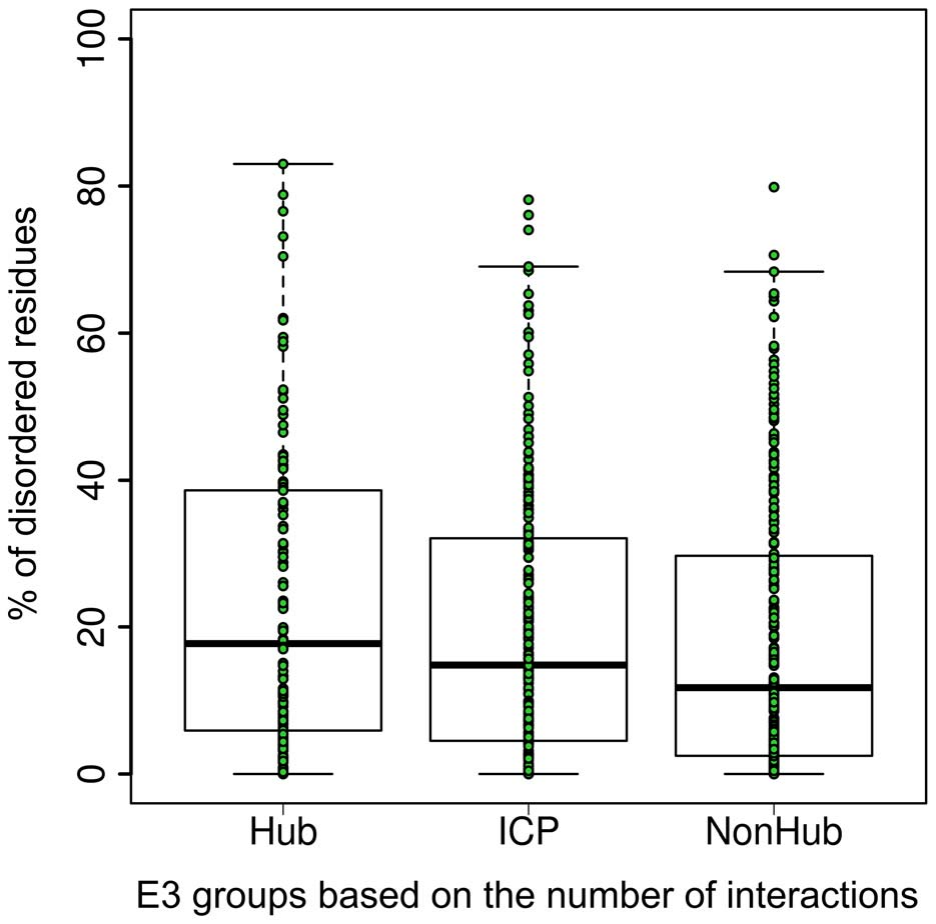
Structural disorder of E3 ligases as a function of their connectivity in the interactome. Disorder content for the three connectivity groups of human E3s (hub: k≥25, ICP: 4≤k≤24, non-hub: k≤3). Green circles represent individual proteins. The bottom and top borders of the boxes represent the 25% and 75% of the data while the bottom and top whiskers indicate 10% and 90% of the data, respectively. The bold line indicates median value.

Due to the frequent involvement and manifold functional advantages of disorder in protein-protein interactions, we next asked if structural disorder in E3s is related to their interaction properties. To this end, we positioned E3 enzymes within intracellular interaction networks by merging two datasets of interaction data: (i) a comprehensive set of experimentally validated binary interactions between a large group of RING-finger/U-box E3 proteins and UBC domain containing E2s [4], and, (ii) interaction data in the STRING database for the 563 E3 ligases. We used the connectivity (‘k’) parameter to classify the E3s into highly connected hubs (H, k≥25), intermediately connected proteins (ICP, 4≤k≤24), and, non-hubs (NH; k≤3), based on the number of their known interaction partners (Table 3 and Tables S2 and S3). Interestingly, even by this high cutoff value (‘k’≥25), almost one fourth of E3s are hubs, which shows the central position they occupy in the interactome. In agreement with earlier general analyses of the relationship between disorder and connectivity [31], [32] hub E3 proteins have the highest mean disorder content (22.9%), which is significantly greater than the corresponding value for the non-hubs (18.4%) (p = 0.009) (Figure 3). Intriguingly, this difference between hubs and non-hubs is even more pronounced in E2-interacting E3s (HECT, U-box, sRF and mRF) (Hubs, 28.02% vs. Non-hubs, 18.68%; p = 0.0015) than in other E3s. A caveat here would be that, so far, not all E3s have been extensively studied experimentally in the context of identifying interaction partners. Therefore, the trends observed here might actually become clearer when further interaction data becomes available.

**Table 3:** Disorder content of E3s and their classification based on their connectivity.

Based on all partners				
	No. of E3s	Avg. length	Avg.% of disordered residues	Avg. no. of interaction partners
HUB	123	621.31	22.9	62.93
ICP	201	733.48	20.27	9.86
Non-HUB	239	612.29	18.36	1

HUBs are significantly more disordered than Non-HUBs: p-value  = 0.009 (22.9 vs 18.36).

Even within single families there is a wide variation, with hubs having much higher disorder than non-hubs (Table S5). Two functional categories – cullins and mRFs – seem to defy this relationship. Members of the mRF family – although two of them are hubs and only one is an ICP – have very low disorder content (0.93% and 0%, respectively for the two classes): they are very short, well-conserved one-(RING)-domain proteins. They are not in direct contact with their substrates and their various E2 and cullin partners in the complexes tend to interact with them in similar ways and using similar interaction sites. Similarly, the cullins are conserved, folded proteins scaffolding multisubunit (msE3) complexes. They do not recognize substrate proteins either: together with mRFs they form a tight complex that serves as the “catalytic center” of msE3s [12], [33], with apparently conventional enzyme-like structural attributes. Not surprisingly, they have rather low disorder content even as hubs or ICPs (4.3% and 14.57% respectively). It is of note that within this basic structural layout, however, there are many possible ways of assembly enabling probably hundreds of different CRL entities [34], which explains their hub status within the interactome.

For proteins that interface the UPS with the proteome (ssE3s and adaptor/substrate recognition subunits of msE3s) structural disorder increases with “hubness” (Table S5). In the HECT, sRF, VHL and SOCS families (which altogether account for 60% of the total number of E3s in our database) there are proteins in all three connectivity groups, and the mean disorder content strictly increases from non-hubs through ICPs to hubs. While for the HECT, sRF and SOCS families the mean disorder content for the hub group ranges between 25–30%, for the VHL family is even higher (>40%). Among the VHL family, only pVHL is a hub by our classification criteria; although only 213 residues long, it has 57 high-confidence interaction partners. In good agreement with its high connectivity, 42% of its residues are disordered. In the ADAP and APC adaptor families all proteins are hubs (mean disorder of 20.6% and 24.02%, respectively). The ADAP, DCAF and F-box family proteins play an adaptor role in the complexes formed by the RBX1 and the RNF7 (RBX2) mRFs, maintaining a bridge between cullin and the actual substrate recognition subunit. Although all these adaptor families contain proteins that are classified into hubs, ICPs and non-hubs, their mean disorder does not always increase as a function of the number of interaction partners. A possible explanation might be the low number of interactions identified thus far, as exemplified by the BTB and F-box families, where a large majority of members are without any known interaction partners, despite their role as an integral part of msE3 complexes (Table S5).

### Structural disorder and E3 function: folding transitions in E3s

**Figure 4:**
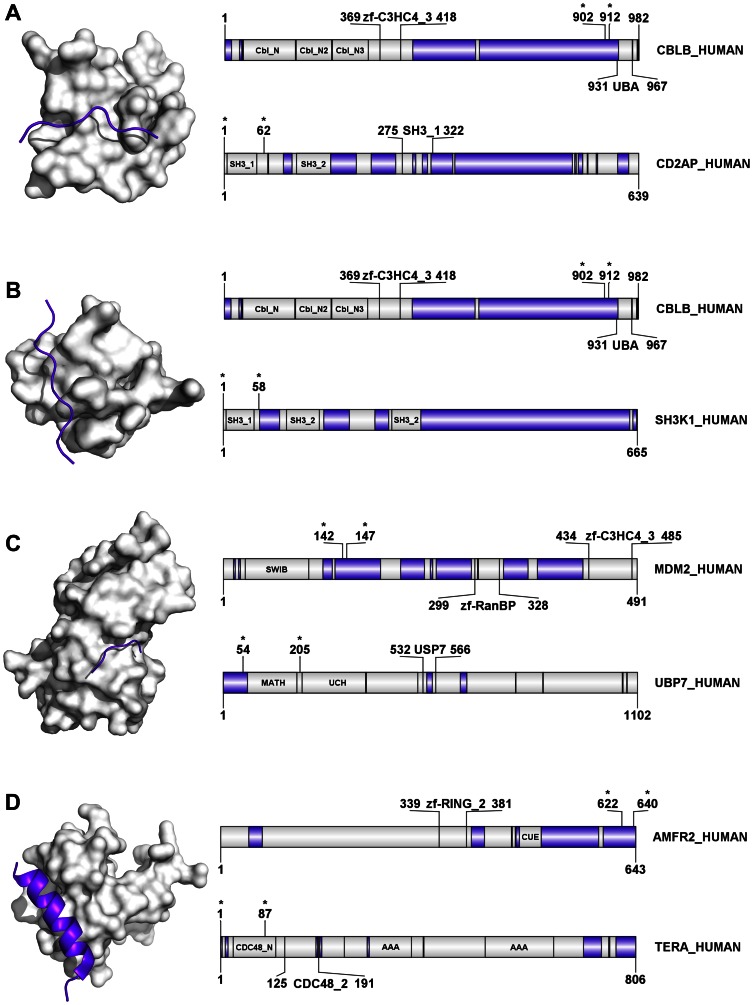
Induced folding of human E3 ligases in interactions with their partner molecules. PDB structures are presented in which a disordered segment of a human E3 ubiquitin ligase binds to the folded domain of a human partner protein (neither an E1/E2/E3 enzyme nor a substrate for the given E3). **A**) Interaction between E3 ligase CBL-B (CBLB) and CD2-associated protein (CD2AP; PDB 2J6F). **B**) Interaction between E3 ligase CBL-B (CBLB) and SH3K1 (SH3 domain-containing kinase-binding protein 1; PDB 2BZ8). **C**) Interaction between E3 ligase MDM2 and UBP7 (Ubiquitin carboxyl-terminal hydrolase 7, also USP7; PDB 2FOP). **D**) Interaction between E3 ligase AMFR2 and TERA (Transitional endoplasmic reticulum ATPase, also VCP; PDB 3TIW). On all four panels the domain maps for the whole chain of both interaction partners are also shown, next to the PDB structure: the upper map is for the E3 ligase, the bottom one is for the partner. In the structures, the disordered E3 chains are represented as purple cartoon while the partner molecule is rendered in surface representation. The domain maps show the lengths and names of the proteins and their domains. The regions predicted to be disordered by IUPred are marked in purple, the ordered segments are in white; the regions present in the PDB structures are delimited by asterisks.

**Figure 5:**
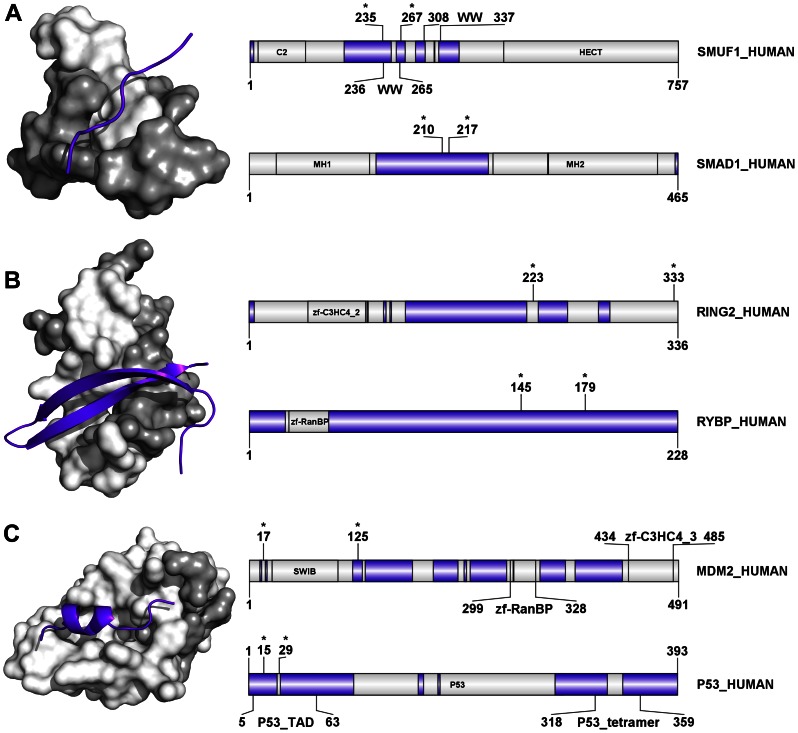
Induced folding in the interaction of E3 ligases and their substrates. Three PDB structures are presented in which induced folding or mutual induced folding (cofolding, synergistic folding) occurs upon interaction of a human E3 ligase with its substrate. **A**) Interaction between E3 ligase SMURF1 and its substrate SMAD1 (SMA and mothers against decapentaplegic homolog 1; PDB 2LAZ) is a case of co-folding of two disordered regions. **B**) Interaction between E3 ligase RING2 and RYBP (RING1 and YY1-binding protein; PDB 3IXS) is also an example of co-folding. **C**) Interaction between E3 ligase MDM2 and P53 (P53 tumor suppressor protein, also TP53; PDB 1YCR), here the substrate undergoes induced folding upon binding to the folded SWIB domain of MDM2. On all three panels PDB structures and domain maps of the two proteins (E3 on top) are shown. On the domain maps, the names of domains, their positions and total length of the protein are indicated. The regions are color coded according to their IUPred disorder status: regions predicted to be disordered are in purple, ordered segments are in light grey. The regions present in the PDB structures are delimited by asterisks. In the PDB structures the disordered segments of partners are shown as purple cartoon whereas the E3 ligase is rendered in surface representation; disordered regions (mapped from disorder predictions on the unbound form) being light grey, and ordered regions white.

Elevated disorder in hub E3s suggests that E3 structural disorder is involved in protein-protein interactions. Often, disordered proteins/regions undergo folding transition upon binding to their partner (induced folding or disorder-to-order transition [35]). To provide concrete evidence that this occurs, we collected 21 non-redundant structures from the PDB in which a human E3 ligase is bound to another human interaction partner. These cases fall into three distinct categories (Table S6): 1) E3 interacting with a UBC domain containing E2 (interaction typically mediated by RING and UBC domains), 2) E3 interacting with (an)other E3 (interaction typically mediated by the RING domains of both proteins), and 3) E3 interacting with proteins other than E2s/E3s: i.e., cofactor, substrate or other miscellaneous partners (interaction mediated by regions other than the RING/U-box/HECT domains). Because structural disorder is potentially involved in this latter category (Table 2), we further analyzed and sub-classified them into four types according to the structural characteristics of the protein segments involved in binding from both partners (Figure 4 and Figure 5): i) ssE3s interacting with their partner via ordered segments of both, ii) induced folding, when the interaction is mediated by a disordered binding region of ssE3 that becomes ordered in the complex, iii) induced folding, when the interaction is mediated by a folded domain of ssE3s and a disordered segment of the partner, and iv) cofolding or mutual synergistic folding [36], when both interacting protein segments are disordered in the unbound state. We outline and analyze the biological functioning of those complexes in which induced folding of a disordered segment takes part in the interaction.

The induced folding of disordered E3 regions is exemplified by CBL-B binding to the SH3 domain of the cofactor CD2AP (Figure 4A) [37], [38]. The same region of CBL-B also binds to the SH3 domain of SH3K1 [39] (Figure 4B), demonstrating the structural adaptability inherent in disordered proteins. The interaction between a disordered segment of the MDM2 ligase and the Math domain of USP7, which contributes to regulating the p53 pathway, is also a case of induced folding of an E3 (Figure 4C). Yet another example of such an interaction is observed between the disordered segment of AMFR2 and the CDC48_N domain of TERA (Figure 4D) [40]. Interestingly, all these interactions that rely on induced folding of E3s occur in complexes with cofactors (CD2AP, UBP7, SH3K1 and TERA) and not with other E1/E2/E3 enzymes or substrates.

Focusing on E3-substrate complexes, the cases we found in the PDB showed that different types of interactions might occur (Figure 5). In two cases, co-folding (mutual folding, synergistic folding) occurs, when both partners are disordered prior to binding to each other. The disordered region of the E3 SMURF1 interacts with receptor-regulated SMADs (SMA and mothers against decapentaplegic homolog, Figure 5A) to trigger their ubiquitination and degradation specifically in the BMP (bone morphogenetic protein) pathway [41]. Co-folding is also apparent between the disordered segment of the E3 RING2 and RYBP (RING1 and YY1-binding protein, Figure 5B), which results in RYBP mono-ubiquitination [42]. The inherent adaptability of IDPs is also demonstrated by the somewhat different molecular logic of E3 MDM2 (murine double minute 2) binding to its premium substrate, p53. As noted above, a disordered segment of MDM2 is involved in binding the co-regulatory USP7 (UBP7_Human, Figure 4C). Here, the disordered segment of p53 binds the folded SWIB domain of MDM2 (Figure 5C). This interaction enhances the AKT-mediated phosphorylation of MDM2 increasing its interaction with p-300 for MDM2-mediated ubiquitination and degradation of p53 [43], [44]. Although these few examples do not enable generalizations, it is at least interesting that in all three cases (p53, RYBP and SMAD1) a disordered region of the substrate is involved in mediating the interaction.

When analyzing the types of secondary structure that the disordered regions (IDRs) adopt in their bound states, we find that out of the four examples of E3-cofactor binding (Figure 4), the AMFR2-TERA interaction alone shows evidence of formation of an α-helical segment (Figure 4D). The other three are no longer disordered, but adopt an extended, coil-like conformation. In the three representative examples of E3-substrate interactions (Figure 5), two of the three cases result in the IDR folding into regular secondary structures (a β-hairpin and a partial α-helix). To understand “induced folding” occurring in these examples from the E3 family, we used the results from a large set of ‘Molecular Recognition Features’ (MoRFs) [45] that characterize those regions of disordered proteins that undergo disorder-to-order transitions upon binding to their partners. Based on the structures adopted after binding, three basic types were described: α-MoRFs, β-MoRFs, and irregular. Nearly 50% of the MoRF dataset consisted of irregular secondary structures. Another previous study had also commented upon the high incidence of coil structures in the bound form of 24 IDPs [46]. Several specific examples of disordered (extended) loop regions in monomeric proteins becoming ‘fixed’ in the interface regions of the complex have also been discussed in the context of disorder-to-order transitions during protein complex formation [47]. Further, this phenomenon may be more universal and not restricted to IDPs; the common occurrence of non-regular secondary structural elements in binding interfaces has also been observed in the case of globular protein-protein interactions, and, in transient hetero-complexes in particular [48].

### Structural disorder and E3 function: the role of inter-domain linkers

**Figure 6:**
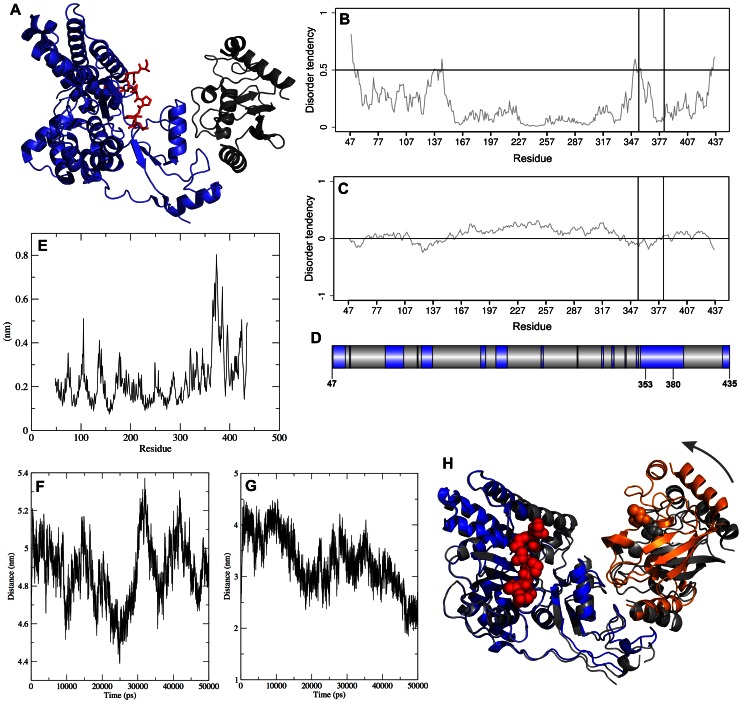
Structural organization and molecular dynamics analysis of an E2-E3-substrate complex. Structural and molecular dynamics analysis of the complex (PDB code: 4A4C) between human CBL, ubiquitin-conjugating enzyme E2, and a peptide derived from the CBL substrate ZAP-70. (A) Structural organization of CBL, as seen in the crystal structure. The E3 molecule is in blue, E2 in dark grey and the ZAP-70 substrate peptide is in red. The predicted disorder profiles of the CBL sequence present in the crystal structure using (B) IUPred, and (C) FoldIndex, respectively. Vertical lines represent the linker helix region (CYS353-CYS381). In the IUPred plot, peaks represent the predicted disordered region(s), whereas in FoldIndex the negative values correspond to unfolded/disordered regions. The disorder calculations were run for the entire CBL sequence (UniProtKB: P22681), but the figure only shows the peptide segment (PRO48 – ASP435) present in the crystal structure. (D) Sequence of CBL with blue color indicating regions with high crystal B-factors (>100Å^2^). (E) RMSF plot from the 50ns MD simulation. (F) Distance between the center-of-masses of the substrate-binding TKB domain of CBL and the E2 as a time-series plot from the MD simulation. (G) Distance between the E2 catalytic CYS and the N-terminal SER of the ZAP-70 peptide. (H) Two orientations (“open” and “closed” forms) of the E2-E3-substrate peptide complex obtained from the NM simulation. They correspond to two extreme configurations (along the lowest frequency normal mode), showing the bending around the linker helix region that acts as a hinge/lever. The “open” configuration is colored dark grey, and the “closed” configuration is colored blue (E3), and orange (E2). The catalytic CYS85 and the substrate peptide are shown in spacefill representation. CYS85 are shown for both the open and closed forms of the structure, whereas the substrate peptide is shown only for the closed form (for clarity). The TKB domains of the two different configurations are structurally superposed using the C-alpha atoms. The TKBD is aligned with very low RMSD, whereas the RING-domain and the E2 have moved significantly in the two conformations (in the direction pointed by the curved arrow).

**Figure 7:**
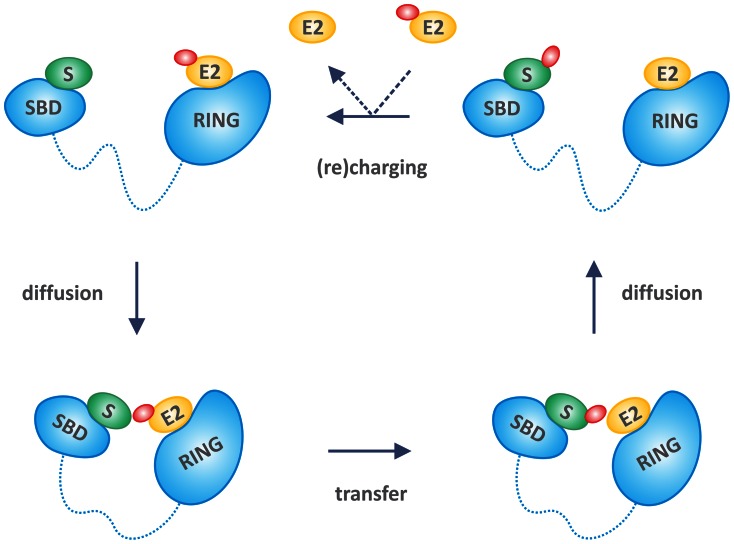
Structural disorder enables intramolecular diffusion in E3 action. A simplified scheme of the linker (entropic chain) function of disordered regions in E3 ligases (for molecular recognition function, see text and Figures 4 and 5). Several ligases of the ssE3 family have a substrate-binding domain (SBD, can also be a disordered motif) and an E2-binding domain (shown as RING here, can be also a U-box or HECT domain) separated by a disordered linker region (dashed line). Due to the conformational freedom of the disordered linker, the bound substrate (S) and ubiquitin-charged E2 (E2∼Ub, ubiquitin shown in red) can diffuse toward and away from each other, without dissociating from the E3. This “intramolecular diffusion” mechanism enables proximity of substrate and E2∼Ub for ubiquitin transfer and also subsequent replacement of E2 with E2∼Ub in a more open conformation, i.e. (re)charging of the ligase. In principle, the flexibility of the linker enables the polyubiquitiniation or multiple monoubiquitination of the substrate, which may explain processivity of the ligation reaction.

To comprehend the linker properties that have evolved in ssE3s, we analysed all sRF and U-box-type ssE3s in our dataset in terms of their UniProt domain assignments. Linkers that connect adjacent E2-recognition and substrate-recognition domains are functionally important for E3 ligase catalytic activity and ubiquitin transfer (a case study is described in detail in the following section). HECT ssE3 family members were not included as these proteins use a very different mechanism for catalysis (as commented upon in the Introduction). For almost one third (91/280) of sRF/U-box E3s, UniProt showed only the presence of a single RING/U-box domain, which means that the substrate recognition is most probably carried out by the surrounding, non-domain regions. The average disorder content of these non-domain regions was ∼30%, implying that, at least in certain cases, disordered regions could be directly involved in substrate recognition. The next scenario (for which a linker region can be clearly identified) involves sRF and U-box ssE3s for which at least one of their domains were previously described in the literature as being capable of substrate recognition. We only considered linkers spanning a RING/U-box domain (binding the E2 with the activated Ub moiety) and an adjacent potential substrate-recognition domain devoid of any intervening other domains or trans-membrane spanning regions. We could identify 90 such linkers in our dataset: an example is shown in Figure 6; see also Figure 7 for a schematic representation). In these cases, the inter-domain linker functions as a flexible hinge bringing these domains into close spatial proximity, thereby facilitating the transfer of ubiquitin from the E2 to the substrate. This dynamic inter-domain motion (Figures 6 and 7) would also potentially account for the processivity observed in ubiquitination and a relatively unrestricted spatial search for the correct ubiquitination site on the target protein (thus enabling poly-ubiquitination, multiple mono-ubiquitination, as well as a variety of other complex Ub-chain extensions).

Linker regions between adjacent E2-binding (RING/U-box) and substrate/adaptor-binding domains were identified. The length distribution of these 90 linkers and their predicted disorder are plotted in Figure S3. 67% (60/90) of the linkers are within 50 residues length, but there are a significant number with lengths between 50–200 and larger than 200 residues (22% and 11%, respectively) (Figure S3A). The longest linker observed in this set was 1576 residues, belonging to the BRCA1 E3 ligase. We also calculated the average disorder score for each linker (Figure S3B): the distribution shows that 37% (33/90) of the linkers have an average disorder score of less than 0.20. However, most of the linkers (63%) have greater than 0.20 average disorder score. Of interest, ∼24% of the linkers have more than 0.40 average disorder score. However, the correlation between linker length and average linker disorder score is poor (correlation coefficient 0.34), indicating that a complex interplay between linker length and disorder may be employed by this family to manage the intricacies of ubiquitination. Moreover, specific E3s could have specific tendencies towards specific chain extension reactions, and this would probably manifest in the properties of the linker; this would also depend on the nature (shape, size and surface properties) of the substrate(s) that the cognate E3 has evolved to recognize and ubiquitinate.

### Case study: large-scale conformational dynamics and E3 ligase activity in human CBL

To demonstrate the potential mechanistic role of highly flexible, disordered linkers in E3 activity, we selected from the PDB the structure of a single-subunit E3 (human Cbl), bound to its cognate E2 and a peptide derived from its substrate ZAP-70 (PDBid: 4A4C) [49]. Cbls are RING ubiquitin ligases that attenuate receptor tyrosine kinase (RTK) signal transduction. The structure of the E3 Cbl consists of an N-terminal tyrosine kinase binding (TKB) domain and a C-terminal RING domain connected by a linker helix region (LHR) (Figure 6A). Cbl ubiquitination activity is stimulated by phosphorylation of a LHR tyrosine residue. We used IUPred and FoldIndex to predict disorder in the E3 sequence. The disorder profiles showed a distinctive peak in the linker region (Figures 6B,C) suggesting that the linker is flexible and therefore might be critical for juxtaposing the E2 and the substrate-binding TKB domains during ubiquitin transfer. The linker in this particular E3 is not an IDR, unlike disordered linkers that may be present in other E3 ligases (Figure S3B). The disorder profile shows a distinctive peak in the LHR (although it does not cross the threshold for an appreciable stretch of residues), and the following analyses also shows this linker to be the most flexible part of the structure, and functionally crucial for the enzymatic activity. The crystal structure of the unbound Cbl (PDBid 2Y1M), however, has missing electron density for the first few residues of the linker, showing that indeed the linker may be at least partially disordered. The profile also shows a second peak in the region 130–145, and this corresponds to an extended surface loop that is part of the substrate-binding domain. When we analyzed the crystal B-factors, again we saw a broad peak in the region encompassing the linker helix (Figure 6D). The domain organization of the E3 molecule and its association with the E2 and the substrate clearly demands that a conformational change altering the relative orientation of the two lobes (TKBD and RING) is required to allow the catalysis to take place, because the distance between the E2 active site Cys residue and the substrate peptide observed in the experimental structure is too large to permit effective ubiquitin transfer. In an attempt to understand and characterize the degree and precise nature of the required conformational change, we applied both normal modes and molecular dynamics simulations to demonstrate the intra-molecular “diffusion” of the E2-binding RING domain and the substrate binding TKB domain towards each other, thereby bringing the ubiquitin and substrate in closer proximity.

A 50 ns molecular dynamics trajectory for the complex was run and analyzed for evidence of linker flexibility. First, we observed high RMSF values around CYS353:CYS381 (the linker helix region) indicating that this region is the most flexible in the entire E3 structure (Figure 6E). During the simulation, the distance between the centers-of-mass of the substrate-binding TKB domain and the E2 fluctuate and at certain times come significantly closer (compared to the distance in the starting crystal structure). We measured the distance between the center-of-masses of the TKBD and the E2 as a function of simulation time, and the plot shows an approximately 1 nm (10Å) fluctuation in the distance (Figure 6F). A similar significant decrease is noticed in the distance between the center-of-masses of the TKBD and the RING-domain of the E3 (that binds directly to the E2) (plot not shown). We also computed the time-series plot of the linear distance between the E2 catalytic CYS and the N-terminal end residue (SER4) of the ZAP-70 substrate peptide (Figure 6G). In the 4A4C crystal structure the E2 catalytic CYS and the ZAP-70 peptide are separated by approximately 28Å. This is a crude approximation for the distance between the catalytic CYS on the E2 and the target LYS residue of the substrate that will be ubiquitinated (not present in the crystal structure); nevertheless the plot shows a dynamic fluctuation in the distance. We note that this linear distance varies between ∼2–4 nm (20–40Å) during the course of the simulation. The minimum distance obtained from the trajectory (∼1.9 nm, or 19Å) lowers the distance observed in the crystal structure to a much more reasonable value for the ubiquitin transfer reaction. Taken together, the MD results clearly indicate an inter-domain closure motion occurring in the E3, with the linker helix region acting as a flexible (also predicted to be disordered using IUPred) hinge.

In order to analyze the long-term dynamical properties of the system, we also examined the normal modes of the complex using the ElNémo webserver. The five lowest frequency modes for the complex were calculated and the motions along each of these specific modes can be visualized as movies showing the structural rearrangements suggested by the Coarse Grained-NMA (Supplementary Zip Files S1). The first and fourth lowest frequency modes in particular appear to enable a long-range conformational change that significantly reduces the linear distance between the E2 catalytic Cys (colored yellow in the supplementary movies) and the substrate peptide (red). The linker helix region (LHR) appears highly flexible and behaves as a swinging lever arm. Thus the normal mode motions clearly identify it as a hinge/lever that enables the relative movement of the E3-RING and the TKBD domains, and is thus responsible for bringing the two domains close to each other. Figure 6H presents the extreme “open” and “closed” forms of the complex taken from the displacement along the lowest frequency normal mode. These low frequency motions readily support the high catalytic efficiency of CBL. To identify the hinge residues, we used HingeProt [50] with the 4A4C PDB structure: two of the three hinge residues in the lowest frequency mode are located in the LHR. Upon repeating the HingeProt analysis using only the E3 ligase coordinates, we found that the sole identified hinge residue in lowest mode 1, and one of the two hinge residues in mode 2 are LHR residues. A comprehensive analysis of such concerted, large-scale rearrangements involving disordered or flexible regions in different types of E3 ligases is currently under progress (Guharoy et al., unpublished results).

## Discussion

The UPS is one of the most important elements of quality control in the cell, maintaining proteostasis, a healthy balance of functional proteins [1], [2]. The system chemically activates ubiquitin via ubiquitin activating (E1) enzymes, which is then transferred to one of a few dozen ubiquitin conjugating (E2) enzymes. E2 with its labile ubiquitin moiety (E2∼Ub) is brought together with the substrate by one of several hundred ubiquitin ligases (E3), which interface the system with the entire proteome. Due to an increasing complexity of the system from ubiquitin to the entire degradome/ubiquitinome within the proteome, we expected an increase in the level of structural disorder from E1 through E3 enzymes. In this study, we observe an overall high structural disorder that increases from E1s to E3s. Although this correlation does not prove involvement in function, there are many individual observations and multiple lines of indirect evidence that substantiate its role in E3s. Due to the extreme heterogeneity and complexity of the system, it is difficult to draw general conclusions; however certain unifying themes clearly appear.

In general, structural disorder is high in proteins having signalling and regulatory roles [13]–[15], where it either provides a flexible link between binding elements (entropic chain function) or it is directly involved in molecular recognition via short binding motifs or domains [16], [17]. In these functions, structural disorder provides many advantages through fine-tuning the kinetics and thermodynamics of molecular recognition events. Based on these premises, the observed elevated level of disorder in E3s is compatible with its use in E3 ligases. Prior limited structural/biophysical studies also demonstrated the abundance (e.g. in MDM2 [19], and BRCA1 [20]), and functional involvement (e.g. in San1 [22], and Sic1 [21]) of structural disorder in E3 action. In case of MDM2, disordered binding motifs (regions 235–259 and 275–289) are involved in the interaction with the highly disordered N-terminal region of Arf, where a mutual binding-induced folding (co-folding) transition occurs, coupled with extensive β-strand formation in both partners [19]. BRCA1 has a more than 1500 residues long disordered region (between domains RING and BRCT; residues 103–1646) that mediates a plethora of different interactions via short peptide motifs showing some secondary structure tendency even prior to binding to the partner [20]. According to Foray et al. [51] BRCA1 acts as a major scaffold protein in DNA damage response binding non-DNA associated downstream phosphorylation targets (such as p53, c-Jun, Nbs1 and Chk2) and enabling ATM or ATR to efficiently modify them.

A unique functional consequence of structural disorder is manifested when two binding elements (motifs or domains) separated by a disordered linker enable a relatively unrestricted spatial search for distinct binding sites. The functional advantages have been described in several well-studied systems, where the linker enhances or even determines specificity [52], enables processivity [53], increases binding strength [54], promotes regulatory communication between distant sites [55], [56], and facilitates the search for distant partners by reaching out in space [57], [58]. In fact, flexibility – without explicitly mentioning structural disorder - is very often mentioned in the E3 literature to explain paradoxical observations, such as the huge gap between the bound E2∼Ub and substrate in CRL (msE3) ligases [8], [9] and processivity in polyubiquitination [5], [59], [60].

In principle, bound E2∼Ub and substrate may be brought together if the two binding regions are linked by a (long) disordered linker region, which enables E3 to undergo large conformational changes between extended and more compact states. For example, this might be the case of MDM2, in which the RING and SWIB domains are separated by 332 residues, and also in BRCA1, in which the RING and BRCT domains are separated by a predominantly disordered region comprising nearly 1500 residues [20]. This kind of mechanism manifests itself even in CRL ligases, which, at first glance, appear as rather rigid complexes [9], [12] presenting a large separation (50–60Å) between bound E2∼Ub and the Lys residue(s) to be modified on the substrate. Whereas a mutation increasing the flexibility of cullin impairs E3 activity [61], it has been suggested that substrate-binding subunits have a flexible inter-domain linker that serves as a hinge, around which the two domains rotate relative to each other to properly position the substrate for ubiquitin transfer [62]. This is also what we demonstrate taking the case study of the single-subunit CBL-B E3 ligase (Figure 6). It was also hypothesized that in the active state of CRLs, E2∼Ub is released from cullin and diffuses toward the substrate. Although this “hit-and-run” mechanism [63] has been criticized [33], it does illustrate clearly the functional opposition between rigidity and flexibility in E3 operation, which may be resolved by structural disorder. Further along this avenue, it was observed that the covalent attachment of the ubiquitin-like NEDD8 protein to cullin stimulates substrate ubiquitination by a special mechanism: X-ray crystallography and SAXS experiments demonstrate that the RING domain of Rbx1 is freed from cullin upon covalent modification by NEDD8, remaining tethered to cullin only by a short linker that can attain multiple conformations [64]. The presence and operation of this dynamic linker is not far from the idea of functionally important structural disorder (“fuzziness”) in the bound state of proteins [65].

Our general premise is that structural disorder between the substrate- and E2∼Ub-binding regions of E3 ligases enables an “intramolecular diffusion” mechanism, in which bound E2∼Ub and substrate are relatively free to move toward and away from each other (Figure 7). This mechanism enables ubiquitin transfer and it would also shed light on yet another mystery of protein ubiquitination, its processivity, which is in stark contrast with the strict geometric restraints of an enzymatic reaction [60]. In quality control, a polyubiquitin chain consisting of at least four subunits is built up by the sequential conjugation of ubiquitin moieties, and, even in regulatory monoubiquitination reactions very often several ubiquitin moieties are attached onto the substrate at adjacent sites (multi-ubiquitination). It was already suggested that structural disorder of the substrate might provide the flexibility necessary to bring adjacent substrate sites in proper orientation [59]. In our model, structural disorder of the E3 itself may enable such intramolecular diffusion, due to which several ubiquitin moieties may be added without full dissociation of the bound substrate (Figure 7). Normal mode simulations have proven effective in representing large-amplitude conformational changes (for example, domain and hinge-bending motions) in proteins [66]. Indeed, it has been shown that for several systems, the lowest frequency modes contribute the most to a conformational change. Although care is required in interpreting the results of molecular simulations, these are extremely relevant for formulating useful hypotheses. In this analysis, we have used state of the art methodologies to gain access to the dynamics of ubiquitin transfer and the role of flexible linker regions in E3 activity. Much of our understanding of the regulation of RING E3s comes from structural and mechanistic studies of multi-protein RING complexes such as cullin-RING ligases (CRLs) [62]. Although both single-subunit and multi-protein classes consist of about 300 members in the human genome, the mechanisms of single-subunit RING E3 regulation remain poorly understood. That is why we have selected the example of human CBL to demonstrate the role of linker flexibility and disorder in the mechanism of ubiquitination. Our results produce a very realistic model that can account for the proposed mechanism of Ub transfer and the manner in which the flexibility of the E3 linker facilitates the functional motion (Figure 6). Function abrogating (and therefore, disease-causing mutations) in c-CBL also point to the importance of the disordered linker for function. Tyr371 of the LHR in c-CBL has emerged as one of the most frequently mutated residues found in people with myeloid neoplasms [67].

The evidence is even more straightforward for the involvement of structural disorder of E3 ligases (or their partners) in protein-protein interactions. The examples collected from the PDB (Figures 4 and 5) demonstrate that binding mediated by induced folding occurs both with cofactors (CD2AP, UBP7, SH3K1 and TERA) and substrates (p53, RYBP and SMAD1). From the substrate side it has been reported that there is a small but significant bias of ubiquitination sites (that lead to degradation for mammalian proteins) to be enriched in disordered regions [68]. Further, the presence of long disordered regions (LDRs) has been shown to be essential for proteasomal degradation in certain studies, with these unstructured regions serving as the initiation region for proteasomal proteolysis [69], [70]. Indeed, the absence of LDRs apparently increases the survival in case of Rad23, and these requirements may reflect a general property of the proteasome [71]. Structural evidence is also provided in many cases that the binding of short disordered degradation motifs (degrons) of E3 substrates occurs via folded protein-protein interaction domains (e.g. WD40 beta propeller or leucine-rich repeat (LRR)) of the substrate recognition subunits of CRLs [72]–[75]. In an extreme case, targeting of yeast Cdk inhibitor Sic1 occurs by binding to the WD40 domain of the Cdc4 subunit of SCF^Cdc4^ through a “polyelectrostatic” interaction [21]. In this largely disordered state, multiple short disordered degrons cooperate in binding, which results in a largely disordered, fuzzy [65] complex between the substrate and its cognate E3.

Although direct structural evidence is missing most of the time, the role of structural disorder in molecular recognition also follows from observations that the binding site falls within a region of E3 that lacks a folded domain. In the founding member of the HECT family, E6-AP, both E6 viral adaptor protein and substrate p53 are bound by a disordered region N-terminal to the HECT domain [76]. The central, 1500-residue long disordered region in BRCA1 has been reported to serve as a scaffold for multiple protein partners (e.g. p53, cMyc) [20]. Ubr1p, which is the E3 component of the N-end rule pathway in yeast, depends largely on a basic region for binding its E2 Ubc2p [77]. The C-terminal Pro-rich and acidic regions of Cbl-C, which is also predicted as extensively disordered, is known to be involved in substrate binding [78]. The most intriguing case is San1, an yeast E3 ubiquitin ligase localized in the nucleus, involved in quality control cellular mechanisms, but with no defined human homologue. San1 can indeed distinguish between the misfolded states of its substrates via intrinsically disordered N- and C-terminal domains [22]. Within these disordered regions, there are short conserved recognition elements, the plasticity of which enables them to transiently bind differently shaped misfolded substrates. Besides E3s, often their partners also use disordered segments for interaction. For example, the E2 Cdc34 uses its long disordered C-terminal domain to bind to SCF [79]. Structural disorder may also be involved in the assembly of CRLs in an even more subtle way. Although E3-E2 interactions are largely mediated by RING-UBC binding, the disordered flanking regions of the UBC domain in family 3 E2 enzymes contributes to the specificity toward E3 partners and also cognate Ub-like molecules [6].

Our comparative studies further provide such indirect evidence for the role of structural disorder in protein-protein interactions in E3 ligases. In the case of msE3s (CRLs), E2∼Ub-, substrate-, and possibly cofactor binding is associated with separate regions/subunits of the complex, all of which are contained within a single polypeptide chain in ssE3s. In agreement with data in the literature [11], our calculations show that 223 out of 302 ssE3s have only one folded protein-protein interaction domain (HECT, RING or U-box), which mediates E2∼Ub binding. Therefore, their binding of additional factors and/or the substrate has to be contributed by (disordered) regions outside the domain. The role of structural disorder in molecular recognition also follows from our interaction network analysis. Structural disorder is known to be significantly higher in proteins of multiprotein complexes and hub proteins with multiple interactions [31], [32]. Similar signs are apparent in the UPS system. First, E3 proteins are by far the most disordered in the network, most likely due to having interaction functions that are more complicated than either E1 or E2 proteins. Second, ssE3s are invariably very disordered (in particular, their substrate- and adaptor-binding regions) (Table 2), whereas subunits of msE3s are much more diverse. msE3 subunits involved in E2∼Ub binding (mRFs) and scaffolding (cullins) are largely ordered (Table 1), whereas subunits with adaptor/substrate recognition functions (e.g. DCAF, BTB, F-box, SOCS…) are as disordered as ssE3s, and often contain long disordered regions. Third, our analysis of hubs based on analyzing the number of interaction partners in the STRING database clearly shows that hub E3 proteins are significantly more disordered than non-hubs (p-value = 0.009) (Table 3).

Besides its prevalence in protein-protein interactions, structural disorder is also abundant in proteins of signaling and regulatory functions [13]–[15], because it enables regulatory communication between remote segments of the protein [56], and also effective regulatory post-translational modifications [16], [80]. These functional modalities also appear in the E3 family. Long-range regulatory communication is apparent in Smurf-2, for example. Smurf-2, a HECT E3 ligase, is kept quiescent by an intramolecular interaction between its N-terminal C2 domain and C-terminal HECT domain, the two domains being separated by a 340-residue, largely disordered stretch interspersed with short WW domains [81]. It is activated by the adaptor Smad7, which displaces the C2 domain by binding to HECT domain and thereby makes it accessible for membrane binding and translocation from the nucleus to the cytoplasm. Regulation by post-translational modifications has also been described in many cases: for example, phosphorylation of a tyrosine in the linker region of Cbl-C results in a more rapid turnover of bound E2 (UbcH5b) leading to activation of E3 activity [82]. A further example is the phosphorylation of MDM2 that relieves autoinhibition, and thereby facilitates the productive interaction of p53 with its SWIB domain [83], [84]. The action of E3s is very often regulated by phosphorylation, where either the E3s themselves undergo modification [8], or their substrates are subject to regulation, for example by the formation of an activated phosphodegron [21], [73].

Two further pieces of evidence attest to the direct and causal involvement of structural disorder in the functioning of E3 ligases. First, the observed scale-free distribution of disorder in this family is a strong indication of this feature (Figure 2A). Scale-free distribution has been observed in many biological networks, such as the number of interaction partners in the interactome [85], and has been interpreted as evidence that strong system-level selection acts on this feature. This selection ensures a relative enrichment for small and large number of occurrences, in comparison to a random distribution, due to their special functional involvement. Here we observe the same behaviour in case of E3s, which strongly argues that their level of structural disorder is a feature subject to strong evolutionary selection forces. An additional indirect evidence for the importance of structural disorder in E3 ligases comes from the location of (familial) missense mutations causing disease. For example, about 10 out of 30 mutations in Parkin [8], and 80 out of more than 100 in BRCA1 [20] occur in disordered regions. Whereas the location or even the type of these mutations reveals little about their exact role, their abundance makes it unquestionable that the disordered regions in which they reside make an essential contribution to the functioning of these E3 ligases.

## Conclusion

We have presented in this work several parallel lines of evidence for the use of structural disorder in the ubiquitination system. Initially, we were intrigued to find scattered in the literature, hints suggesting the existence and use of disorder in this system (as detailed in Discussion). In the present study, in order to formalize the role of disorder, we collect all currently available information about the sequences, interactions and structural data for Ub-enzymes, and then describe the occurrence and location of disorder in the context of their sequences, structures and interactions. We find that the E3 protein family exhibits significantly higher disorder characteristics than the other members. In the pyramidal setup of the Ub-system (where the E3s can be seen to form the connecting bridge between the UPS and the proteome), this unambiguously indicates that structural disorder confers manifold functional advantages in E3 function. The E3/substrate interface is also functionally the most complex, as it entails recognition of many thousands of potential substrates by approximately 600 human E3 ligases (meaning that a particular E3 would be responsible for ubiquitinating multiple substrates). In accordance with these requirements, we indeed observe that the E3 system is critically dependent upon disorder features that principally enable (i) multi-specificity partner (substrate) recognition, and, (ii) E3 catalytic function (ubiquitin transfer to substrate) and its inherent processivity. The different types of evidence presented in this work include bioinformatics predictions of structural disorder, disorder in the context of structural data for E3-substrate/cofactor/adaptor combinations, and molecular dynamics-based mechanisms of action of E3 ligases involving flexible (and, sometimes, predicted disordered) linkers. These merge together to present a comprehensive picture of the manner in which structural disorder facilitates the mode of action of ubiquitinating enzymes.

## Methods

### Downloading human E1, E2 and E3 data from the KEGG BRITE database

Two well-annotated human E1 sequences were extracted from the KEGG BRITE database [23]–[25] (Table S1). Of the 30–40 E2s predicted for the human genome [86], 35 known to be involved in ubiquitination were obtained from the literature [4]. E2 sequences were also downloaded from the KEGG database: 33 sequences were obtained, but all these E2s were already present within the literature set. In order to identify redundant sequences, we ran the CD-HIT clustering algorithm with a threshold of 85% sequence identity. 29 sequences (out of the 35) were kept following this filter, by always retaining the best annotated one from the clusters of highly similar sequences (Table S1).

We retrieved a total of 468 proteins from the KEGG BRITE database that we grouped as “E3” components. Those included HECT, RING, U-box (and RBR, within the RING group) E3s together with their putative scaffolding, adaptor, substrate-recognition, accessory and/or regulatory proteins (see Table S7). This initial list of E3-components was screened to obtain a high-quality and reliable dataset (several filtering criteria were applied). In the first step, we removed four KEGG sequences with ambiguous and uncertain annotations such as “acting like”, “by similarity”, “potential”, “probable” or “possible”. Second, we checked if there were multiple KEGG entries with identical gene names: only one such instance was observed (KEGG HSAs 51130 and 100302652 had an identical UniProt gene name ‘ASB3’), and only the “reviewed” (i.e., manually annotated in UniProt) sequence (HSA: 51130) was retained out of the two [87]. Third, one single-RING-finger (sRF, belonging to ssE3s) E3 (HSA: 390231) was found to be a pseudogene, and fourth, we identified another candidate E3 (HSA: 652346) for which there was no sequence information in the KEGG database; these two were removed from our dataset. Finally, the same sequence identity filtering was run (as described earlier for the E2 dataset): in this step, 10 proteins with more than 85% identity to another better annotated protein in the dataset were deleted. At the end of all these steps, 451 E3 sequences remained. The classification of E3s into families was adopted from the literature and KEGG BRITE database [11], [23], [24] (Table S8, cf. also Table 1).

### Collecting human E3 data from the literature

Based on sequence similarity criteria and the presence of characteristic domain signatures, 617 E3 proteins have been identified in the human genome so far [11]. 309 of these are RING finger/U-box proteins, of which only 250 are well studied experimentally [4]. Out of these 250 high-confidence RING/U-box E3s, we could successfully assign UniProt IDs to 249 E3s. Next, identical proteins (from amongst these 249) were removed by filtering for identical UniProt IDs, and 240 unique UniProt IDs were retained. Since we were interested only in human proteins, we replaced one rat protein with its human homologue and deleted three mouse proteins (with no identified homologues in human). We also deleted three further entries because their IDs were removed from UniProt since publication of the van Wijk et al [4] collection of E3 proteins. Finally, we used the CD-HIT algorithm to remove sequences above 85% sequence identity. Application of all these filtering criteria resulted in 219 well-annotated human RING finger/U-box E3 proteins (Table S9).

### Merging E3 proteins retrieved from KEGG database and literature

We felt the need to create a carefully annotated (and updated) dataset of human E1, E2 and E3s. Although the previously published list of 617 predicted E3s [11] is considered a classical paper in the field, not all of those proteins have been characterized as *bona fide* E3s (particularly, as some of them do not have detectable binding to any E2). Therefore, in this work, we attempt to bridge the gap between predicted E3 sets (compiled on the basis of sequence and structural homology matches), and known experimental evidence from the literature and also from databases that employ manual curation. The basic difference between the two parallel E3 datasets (described in the two earlier sections) is that data in the KEGG BRITE database [23]–[25] are manually curated based on experimental information in the literature, whereas the dataset based on reference [4] contain predicted E3 ligases identified based on sequence similarity/motif patterns. Therefore, to obtain a single, comprehensive and well-annotated database of human E3 proteins, we decided to carefully merge these two datasets (Figure S1). Of the 161 and 212 sRFs from the KEGG and literature-based [4] sets respectively, 91 were in common. All 4 U-box and 3 mRF proteins in the literature set were also found in KEGG. After merging, we repeated the 85% sequence identity filtering, which identified 9 highly similar sequences; these were removed. To summarize, our composite dataset contained sequence data for 305 HECT/RING-finger/U-box E3s (302 ssE3s and 3 mRFs) and 258 adaptor/substrate recognition E3s (563 in total; for the number in different families, cf. also Table 1 and Figure 1), alongside 2 E1 and 29 E2s (Table S2 and Table S3).

### Prediction of structural disorder

We used the IUPred method [88], [89] for predicting structural disorder in all the sequences in our database. IUPred returns a disorder score between 0.0 and 1.0 for every residue in the sequence; a value ≥0.5 indicates local structural disorder. From residue-specific scores, we calculated several global measures of disorder for the proteins, such as the number and ratio of their disordered residues (the latter referred to as disorder content), and the length of their longest consecutive disordered segment. We also counted proteins, which have at least one long disordered region (≥30 consecutive residues) ignoring short intervening ordered regions not longer than three consecutive residues. Proteins were considered mostly ordered (O) if the ratio of their disordered residues was less than 0.5; otherwise they were considered mostly disordered (D) (Tables S1, S2 and S3). To calculate the mean of any of the measures determined, we averaged the individual values without using any weighting. We also re-calculated the disorder content using two other standard predictors, FoldIndex [27] and DisProt-VSL2 [90], to test if (and to what extent) the results depend on the nature of the predictor(s) used. Unless explicitly specified, the predicted disorder results correspond to calculations performed using IUPred.

### Interaction classification

We collected interaction data for all 563 E3s from two different sources (Tables S2 and S3). First, we used the results of a large-scale analysis of binary interactions between RING finger/U-box E3s and UBC domain containing E2s [4] which reported physical interactions between 104 E3s and 20 E2s (Table S9). Second, we extracted known interactions from the STRING database for all E3s [91] with a confidence score set to 0.7 (high confidence interactions). We studied the connectivity (‘k’) of E3s and grouped them as hubs (H, k≥25), intermediately connected proteins (ICP, 4≤k≤24) and non-hubs (NH; k≤3) [92] based on the number of their reported interaction partners (Tables S2 and S3).

### Collection of structural information on E3 interactions

We collected all the distinct (by 95% sequence identity filter) structures from the PDB database [93] in which a human E3 ubiquitin ligase is in complex with any other human protein. The interactions found are listed in Table S6.

### Definition of linker regions in sRF and U-box E3 ligases

We downloaded the complete UniProt annotation for the 280 sRF and U-box E3 ligases present in our database. 36 proteins were excluded from the linker analysis due to the lack of any RING/U-box domains in their UniProt feature table annotation. In case of 91 E3s the RING/U-box domain was the only domain annotated by UniProt; since the region responsible for substrate recognition could not be defined precisely in these proteins, they were discarded from the linker analysis. For the remaining proteins the non-RING/U-box domains were collected and they were categorized as potential substrate recognition or non-substrate recognition domains based on extensive literature mining and information provided by protein domain family databases. 23 potential substrate recognition domains were identified. All those regions were accepted as linkers, which are located between a RING/U-box domain and an adjacent potential substrate recognition domain and are not interrupted by any other domain or transmembrane region. At the end 90 such linkers could be identified, which were subject to length distribution and disorder content analysis.

### Tests of statistical significance

Mann-Whitney U-tests were used for calculating statistical significance of observed differences between different groups. This test was applied because it is a non-parametric test that does not make any assumptions about the normality of the datasets being compared. The implementation as available in the R package (http://www.R-project.org/) was used. Comparisons between E1s and the other classes were not performed because there were only 2 E1 proteins. Within the E3 sub-classes, the U-box and mRF families were also not used for statistical comparisons because of the paucity of family members (6 and 3 proteins, respectively).

### Normal-mode analysis

Normal mode analysis was performed on the PDB structure 4A4C. This multi-protein complex comprises the tyrosine kinase binding (TKB) domain, linker helix region and the RING domain of a single-subunit E3 (human CBL), bound to its cognate E2 (UbcH5B) and a 12-residue peptide derived from the CBL substrate ZAP-70 tyrosine kinase. Coarse-grained normal-mode analysis (NMA) was performed using the *elNémo* web server [94]. The coarse-grained elastic network model provides reliable descriptions of long-range, concerted conformational dynamics [95]. In this approach the concerted motions are calculated within the quasi-harmonic approximation of the free energy around a protein's native state (assumed to coincide with the energy-minimized model obtained from the starting crystal structure). The RTB (rotations-translations of blocks) approach [96] implemented in elNémo was used. This construction represents each residue as a rigid block, and translations/rotations between blocks defines the motions of the system. Eliminating the first six frequencies (corresponding to three rotational and three translational movements of the whole system), we studied the CG-NMA results from the lowest five non-trivial modes. To overcome potential biases due to starting from a single initial configuration, the ENM was built and the normal modes calculated using three different conformations (the initial x-ray configuration, and two different snapshots selected from a MD simulation (see following section)). The two MD snapshots (at 32 and 25 ns) used for repeating the NMA correspond respectively to an “open” and a “closed” structural state of the complex as obtained from a clustering of the structure configurations from the entire MD trajectory.

### Molecular Dynamics simulation

Even though normal mode calculations are powerful in obtaining long-range movements such as inter-domain motions, molecular dynamics (MD) simulations are useful to have atomic-level details. Therefore, we performed all-atom, explicit solvent MD simulation of the 4A4C protein complex. First, an incomplete residue (Chain C: Thr129) of a surface loop was built into the structure using the SuperLooper prediction server [97], and missing side chain atoms were modeled using the WHAT IF server [98]. Next, molecular dynamics (MD) simulations were performed with the Gromos96 43a1p [99] force field as implemented in GROMACS 4.5.4 [100]. This forcefield includes entries for phosphorylated residues, which were required as the 4A4C structure includes two phospho-tyrosine residues, including pTYR 371 on the CBL ligase, that functions as an important conformational switch and primes the E3 for catalysis [49]. The models were solvated using simple point charge water molecules in a cubic box with a minimum distance of 10Å from the edge of the box to any protein atom. Adding chloride ions neutralized the net charge of the system. To eliminate unfavorable contacts and steric overlaps, the solvated system was minimized using the steepest-descent method. Then the system was heated from 0 to 300 K in 100 ps constraining protein atoms to allow for the relaxation of solvent molecules. Production simulations were performed for 50 ns with the NPT ensemble at 300 K and room pressure. Temperature and pressure were controlled using the modified Berendsen thermostat [101] and Parrinello-Rahman barostat respectively, as implemented in Gromacs. The system was simulated under periodic boundary conditions with cutoffs of 10 Å each for electrostatic and van der Waals terms. The long-range electrostatic interactions were calculated with Particle Mesh Ewald summation. Initial velocities were generated randomly from a Maxwell distribution at 300 K in accordance with the masses assigned to the atoms. During the production runs, a time step of 2 fs was used in the Leapfrog algorithm, and the LINCS algorithm [102] was used to constrain all bond lengths except those in water molecules. Simple harmonic distance restraints to the coordinating residues were applied to keep the metal ions (2 Zinc and 1 Calcium) in their correct positions in the structure. Coordinates were recorded every 2 ps. Structural alignments and figure rendering were performed using PyMol [103].

## Supporting Information

Table S1 Contains information regarding E1s and E2s and all disorder related calculations performed
(XLS)

Table S2 All information regarding 305 E3s, for which data have been collected from the literature and KEGG database, and also all calculated data related to disorder and connectivity are included.
(XLS)

Table S3 All information regarding 258 adaptor proteins, for which data have been collected from KEGG database and all calculated results, related to disorder and connectivity are included.
(XLS)

Table S4 Mean disorder content for E1, E2 and E3 families predicted using three different predictors.
(DOC)

Table S5 Mean disorder content and connectivity level for Hubs, intermediately connected proteins and NonHubs for different families of E3s.
(XLS)

Table S6 List of PDB structures showing E3 ligase interactions with human partners.
(DOC)

Table S7 Whole set of E3s downloaded from KEGG.
(DOC)

Table S8 Collection of well-studied E3s from KEGG.
(DOC)

Table S9 All E3s obtained from the literature.
(DOC)

Figure S1 **Merging E3 datasets obtained from KEGG-BRITE database and literature.** Schematic illustration of the merging of the different categories of E3 ligases obtained from the KEGG-BRITE database and by literature mining. The principal categories of E3 proteins are shown at the top, and the number collected for each category provided below (blue and red circles represent the number of proteins extracted from KEGG and literature sources, respectively). The number of proteins common between the two sets is shown within the intersecting region. The second row of circles shows the number of proteins in each group after merging the datasets. All proteins in each category are then pooled together, followed by the 85% sequence identity filtering, to obtain the final set of 563 E3 enzymes (detailed description of each step is provided in the Methods section).
(TIF)

Figure S2 **Analysis of long disordered regions (LDRs).** (A) Fraction of disordered residues present within Long Disordered Regions (LDRs). This value is calculated as n_LDR_/N_tot_, where, n_LDR_ is the number of residues present within LDRs, and N_tot_ is the protein length. This ratio (expressed as a percentage) is calculated for each protein, and the distribution is plotted here. The bars represent E3 ligases, whereas the smooth line represents the data for the human proteome (as done in Figure 2A). (B) Abundance of LDRs in E3 ligases (compared to the occurrence of LDRs in the human proteome). The final bin in this histogram corresponds to proteins with 10 or more LDRs within their sequence.
(TIF)

Figure S3 **Analysis of properties of inter-domain linkers linking adjacent E2-binding and substrate/adaptor binding domains in RING and U-box ssE3s.** (A) Length distribution (last bin corresponds to 3 proteins being longer than 450 residues). (B) Average disorder score in linker region (Scores calculated by IUPred).
(TIF)

Supplementary Zip Files S1 Animated gif image files (labelled 4A4C_nm1.gif to 4A4C_nm5.gif) showing the normal mode transitions along the lowest frequency normal modes 1 to 5. In these movies, the E3 ligase (c-CBL) is in blue cartoon representation, the E2 in grey colored surface representation (with the catalytic CYS85 in yellow), and the substrate peptide in red VDW representation.
(ZIP)
