# Supplementary material for: Functional Diversity and Structural Disorder in the Human Ubiquitination Pathway
Source: PLoS One. 2013 May 29;8(5):e65443. doi: 10.1371/journal.pone.0065443 (PMC3667038; doi:10.1371/journal.pone.0065443)
Supplement: Table S4 — Mean disorder content for E1, E2 and E3 families predicted using three different predictors. (DOC) [file pone.0065443.s004.doc]

|  | Avg disorder % | | |
| --- | --- | --- | --- |
| Enzyme Family (number of members) | **IUPred** | **VSL2** | **FoldIndex** |
| E1(2) | 5.97 | 18.10 | 20.10 |
| E2(29) | 17.74 | 37.51 | 32.90 |
| E3(563) | 20.03 | 39.59 | 33.02 |
