# Supplementary material for: Functional Diversity and Structural Disorder in the Human Ubiquitination Pathway
Source: PLoS One. 2013 May 29;8(5):e65443. doi: 10.1371/journal.pone.0065443 (PMC3667038; doi:10.1371/journal.pone.0065443)
Supplement: Table S6 — List of PDB structures showing E3 ligase interactions with human partners. (DOC) [file pone.0065443.s006.doc]

| **PDB ID** | **E3 ligase** | **Uniprot ID** | **Partner** | **Uniprot ID** | **Biological Unit** |
| --- | --- | --- | --- | --- | --- |
| **E3-E2 COMPLEXES** | | | | | |
| 1C4Z | UBE3A(HECT) | Q05086 | UBE2L3 | P68036 | TETRAMERIC |
| 2YHO | MYLIP(SRF) | Q8WY64 | UBE2D1 | P51668 | DIMERIC |
| 3H8K | AMFR(SRF) | Q9UKV5 | UBE2G2 | P60604 | DIMERIC |
| **E3-E3 COMPLEXES** | | | | | |
| 2H0D | BMI1(SRF) | P35226 | RNF2(SRF) | Q99496 | DIMERIC |
| 2VJE | MDM2(SRF) | Q00987 | MDM4(SRF) | O15151 | TETRAMERIC |
| 3DPL | RBX1(MRF) | P62877 | CUL5(ADAP) | Q93034 | DIMERIC |
| **ALL OTHER E3 COMPLEXES** | | | | | |
| 2KXQ | SMURF2(HECT) | Q9HAU4 | SMAD7 | O15105 | DIMERIC |
| 2J6F^*^ | CBLB(SRF) | Q13191 | CD2AP | Q9Y5K6 | DIMERIC |
| 2FOP^*^ | MDM2(SRF) | Q00987 | USP7 | Q93009 | DIMERIC |
| 2BZ8^*^ | CBLB(SRF) | Q13191 | SH3KBP1 | Q96B97 | TRIMERIC |
| 3TIW^*^ | AMFR(SRF) | Q9UKV5 | VCP | P55072 | DIMERIC |
| 2IWG | TRIM21(SRF) | P19474 | IGHG1 | P01857 | TETRAMERIC |
| 2A25 | SIAH1(SRF) | Q8IUQ4 | CACYBP | Q9HB71 | TETRAMERIC |
| 2LAZ^*¥^ | SMURF1(HECT) | Q9HCE7 | SMAD1 | Q15797 | DIMERIC |
| 3ASK^¥^ | UHRF1(SRF) | Q96T88 | H3F3A | P84243 | DIMERIC |
| 3BUX^¥^ | CBL(SRF) | P22681 | MET | P08581 | DIMERIC |
| 3DB3^¥^ | UHRF1(SRF) | Q96T88 | HIST2H3A | Q71DI3 | DIMERIC |
| 3IXS^*¥^ | RNF2(SRF) | Q99496 | RYBP | Q8N488 | DIMERIC |
| 2Y1N | CBL(SRF) | P22681 | ZAP70 | P43403 | DIMERIC |
| 3U5N^¥^ | TRIM33(SRF) | Q9UPN9 | HIST1H3A | P68431 | DIMERIC |
| 1YCR^¥^ | MDM2(SRF) | Q00987 | TP53 | P04637 | DIMERIC |

The PDB entries where the segment of the E3 ligase taking part in the interaction is predicted to be disordered in the unbound form are marked by *, if the segment of the partner is disordered it is marked by ^¥^. In case of the 2KXQ structure, the partner protein was not clearly defined in the PDB database; we identified SMAD7 to be the partner molecule by BLAST search against the human entries of the UniProt database. The last 15 residues of the 20-residue partner molecule where found in SMAD7 and this gave the best hit, so we accepted it as the partner.
