# Supplementary material for: Functional Diversity and Structural Disorder in the Human Ubiquitination Pathway
Source: PLoS One. 2013 May 29;8(5):e65443. doi: 10.1371/journal.pone.0065443 (PMC3667038; doi:10.1371/journal.pone.0065443)
Supplement: Table S8 — Collection of well-studied E3s from KEGG. (DOC) [file pone.0065443.s008.doc]

| **Family** | **Number of identified E3s** |
| --- | --- |
| **Single Subunit E3s** | |
| **Ubox** | 6 |
| **Hect** | 22 |
| **Single Ring Finger (sRF)** | 161 |
| **Multi subunit E3s and their accessory E3 partners** | |
| **Multi subunit Ring Finger (mRF)** | 3 |
| **ADAP** | 4 |
| **APC** | 9 |
| **CUL** | 6 |
| **VHL** | 6 |
| **DCAF** | 23 |
| **BTB** | 105 |
| **FBOX** | 71 |
| **SOCS** | 35 |
