# Supplementary material for: Functional Diversity and Structural Disorder in the Human Ubiquitination Pathway
Source: PLoS One. 2013 May 29;8(5):e65443. doi: 10.1371/journal.pone.0065443 (PMC3667038; doi:10.1371/journal.pone.0065443)
Supplement: Table S9 — All E3s obtained from the literature. (DOC) [file pone.0065443.s009.doc]

| **Family** | **Number of identified E3s** |
| --- | --- |
| **Classification based on domain composition** | |
| **Single Ring finger** | 212 |
| **Ubox** | 4 |
| **Multi Subunit Ring finger** | 3 |
| ***Total*** | ***219*** |
