# Supplementary figures and images for: Functional Diversity and Structural Disorder in the Human Ubiquitination Pathway
Source: PLoS One. 2013 May 29;8(5):e65443. doi: 10.1371/journal.pone.0065443 (PMC3667038; doi:10.1371/journal.pone.0065443)

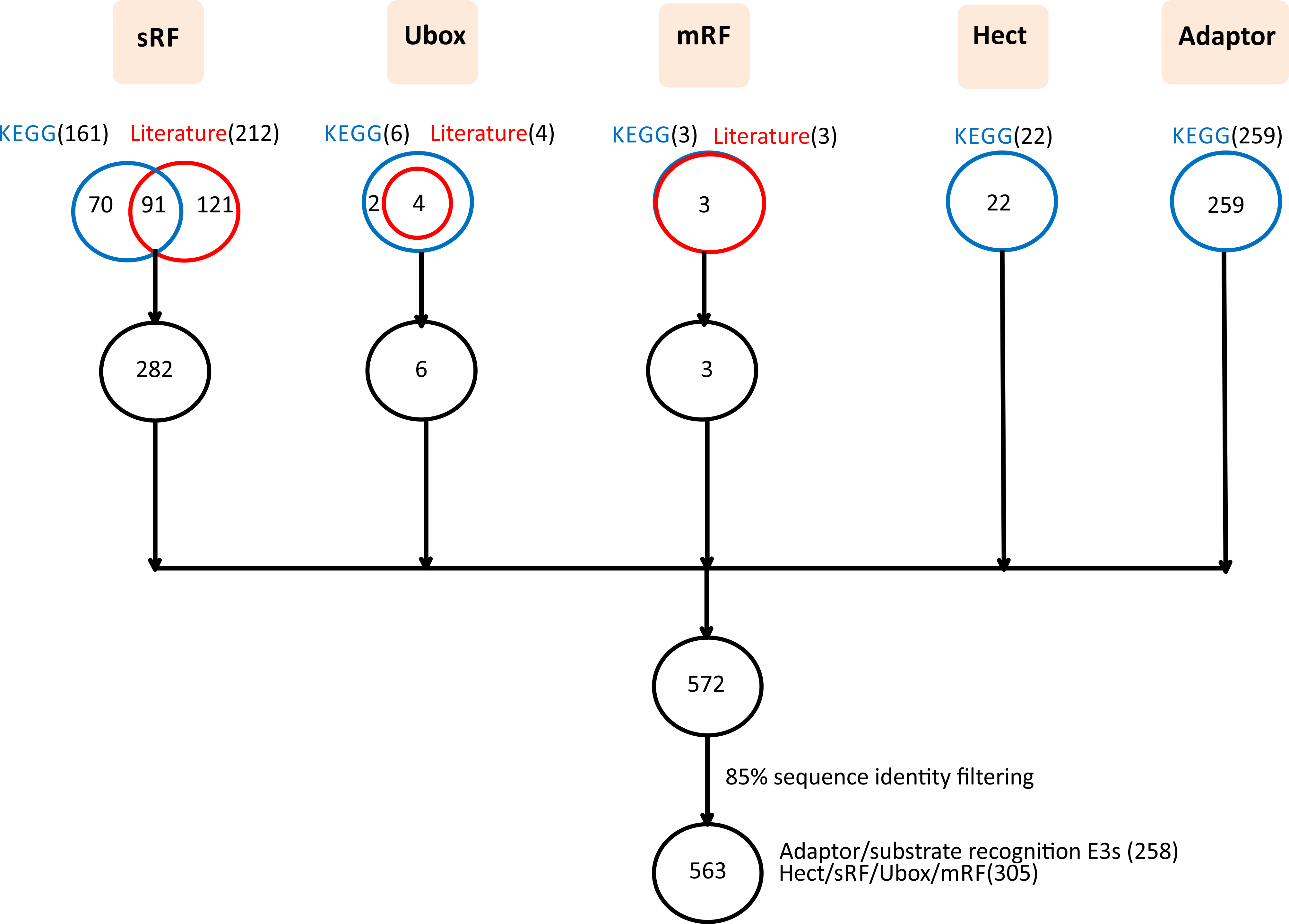

Supplement: Figure S1 — Merging E3 datasets obtained from KEGG-BRITE database and literature. Schematic illustration of the merging of the different categories of E3 ligases obtained from the KEGG-BRITE database and by literature mining. The principal categories of E3 proteins are shown at the top, and the number collected for each category provided below (blue and red circles represent the number of proteins extracted from KEGG and literature sources, respectively). The number of proteins common between the two sets is shown within the intersecting region. The second row of circles shows the number of proteins in each group after merging the datasets. All proteins in each category are then pooled together, followed by the 85% sequence identity filtering, to obtain the final set of 563 E3 enzymes (detailed description of each step is provided in the Methods section). (TIF) [file pone.0065443.s010.tif]

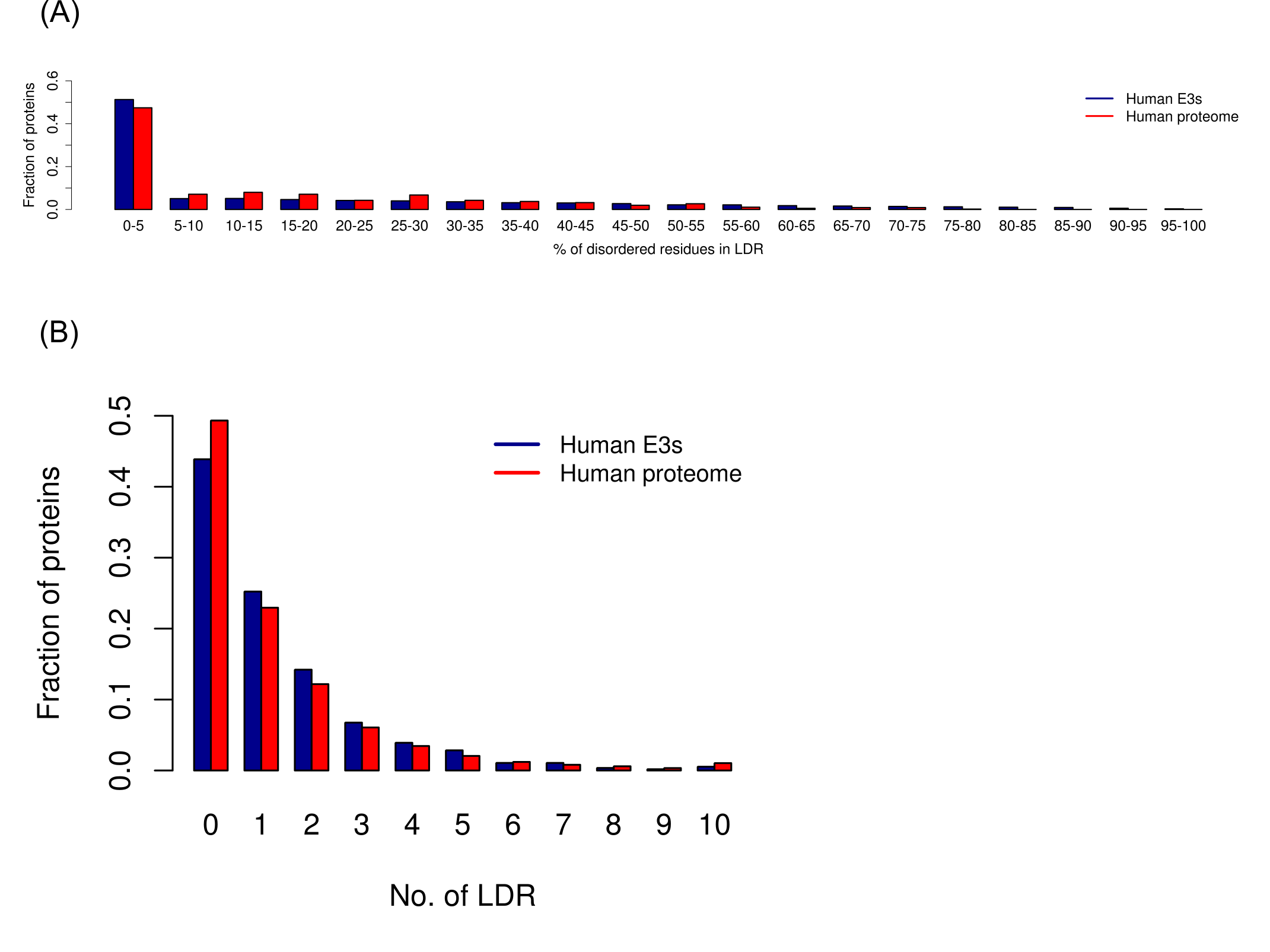

Supplement: Figure S2 — Analysis of long disordered regions (LDRs). (A) Fraction of disordered residues present within Long Disordered Regions (LDRs). This value is calculated as nLDR/Ntot, where, nLDR is the number of residues present within LDRs, and Ntot is the protein length. This ratio (expressed as a percentage) is calculated for each protein, and the distribution is plotted here. The bars represent E3 ligases, whereas the smooth line represents the data for the human proteome (as done in Figure 2A). (B) Abundance of LDRs in E3 ligases (compared to the occurrence of LDRs in the human proteome). The final bin in this histogram corresponds to proteins with 10 or more LDRs within their sequence. (TIF) [file pone.0065443.s011.tif]

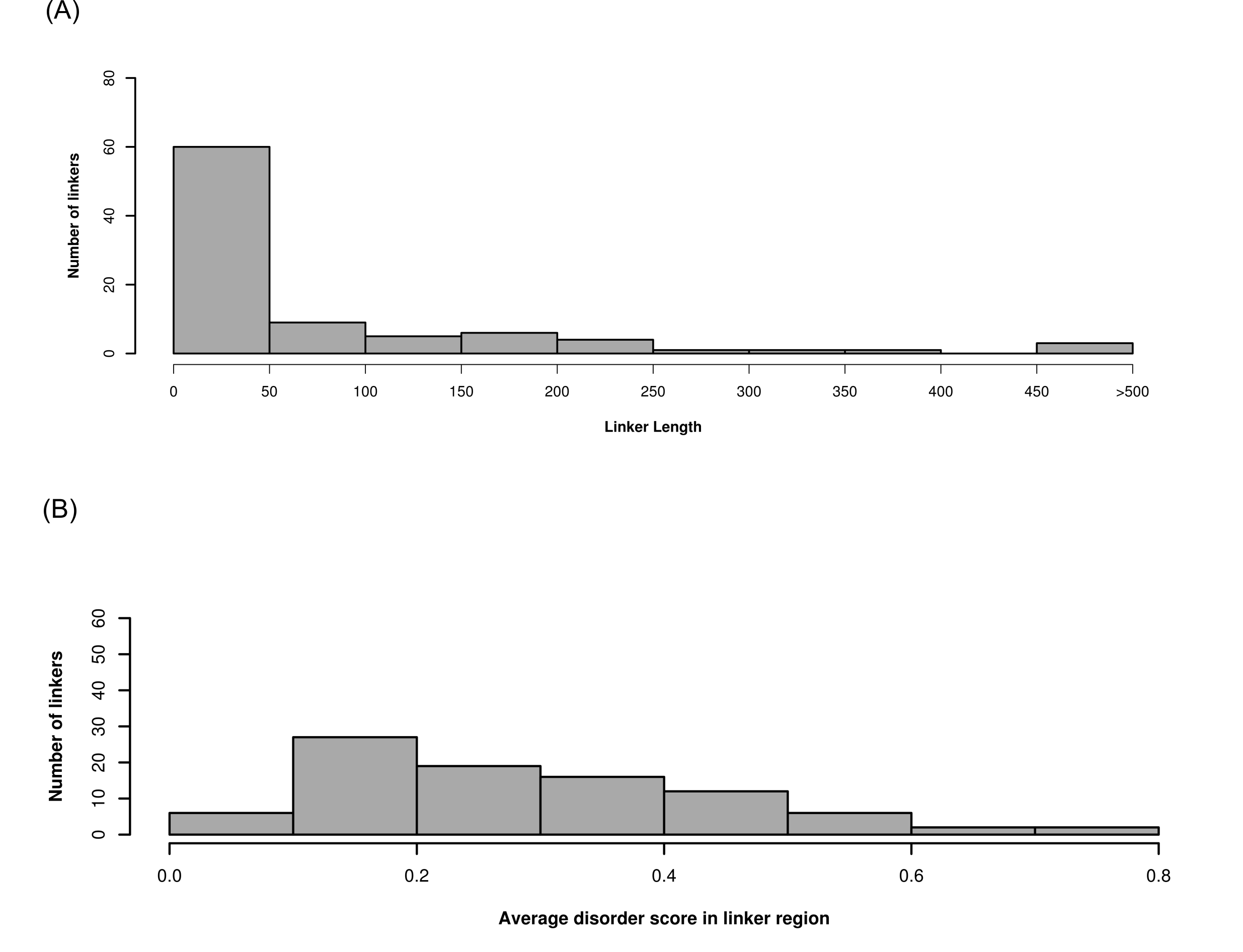

Supplement: Figure S3 — Analysis of properties of inter-domain linkers linking adjacent E2-binding and substrate/adaptor binding domains in RING and U-box ssE3s. (A) Length distribution (last bin corresponds to 3 proteins being longer than 450 residues). (B) Average disorder score in linker region (Scores calculated by IUPred). (TIF) [file pone.0065443.s012.tif]

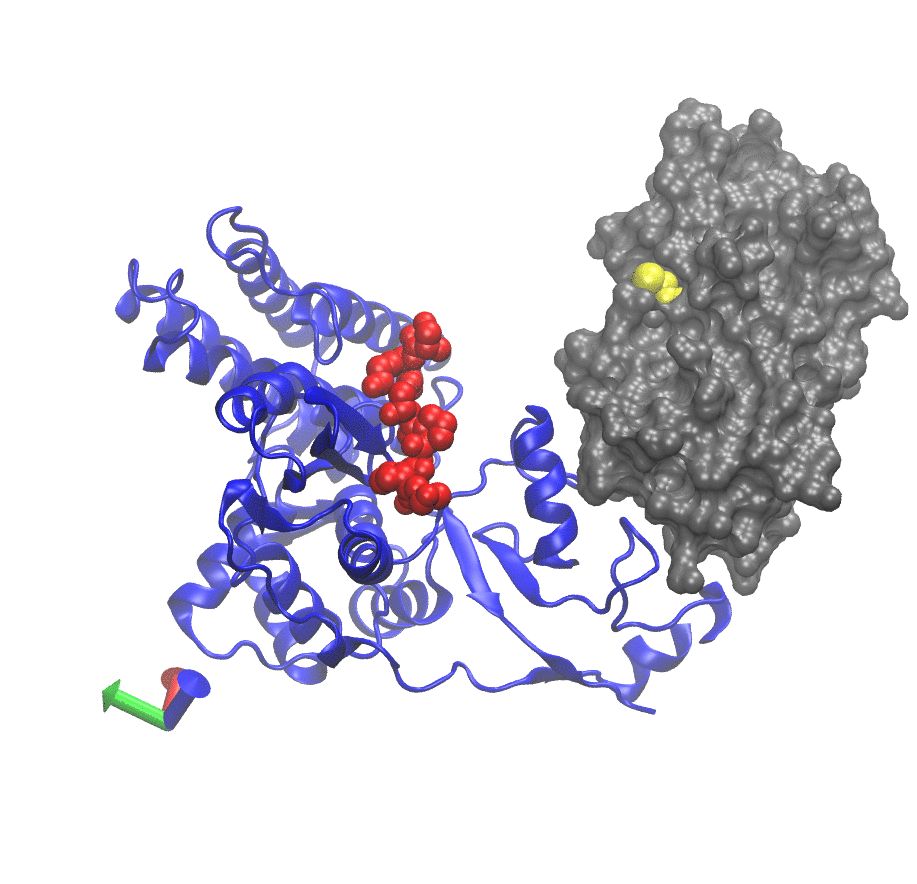

Supplement: Supplementary Zip Files S1 — Animated gif image files (labelled 4A4C_nm1.gif to 4A4C_nm5.gif) showing the normal mode transitions along the lowest frequency normal modes 1 to 5. In these movies, the E3 ligase (c-CBL) is in blue cartoon representation, the E2 in grey colored surface representation (with the catalytic CYS85 in yellow), and the substrate peptide in red VDW representation. (ZIP) [file pone.0065443.s013.zip › 4A4C_nm1.gif]

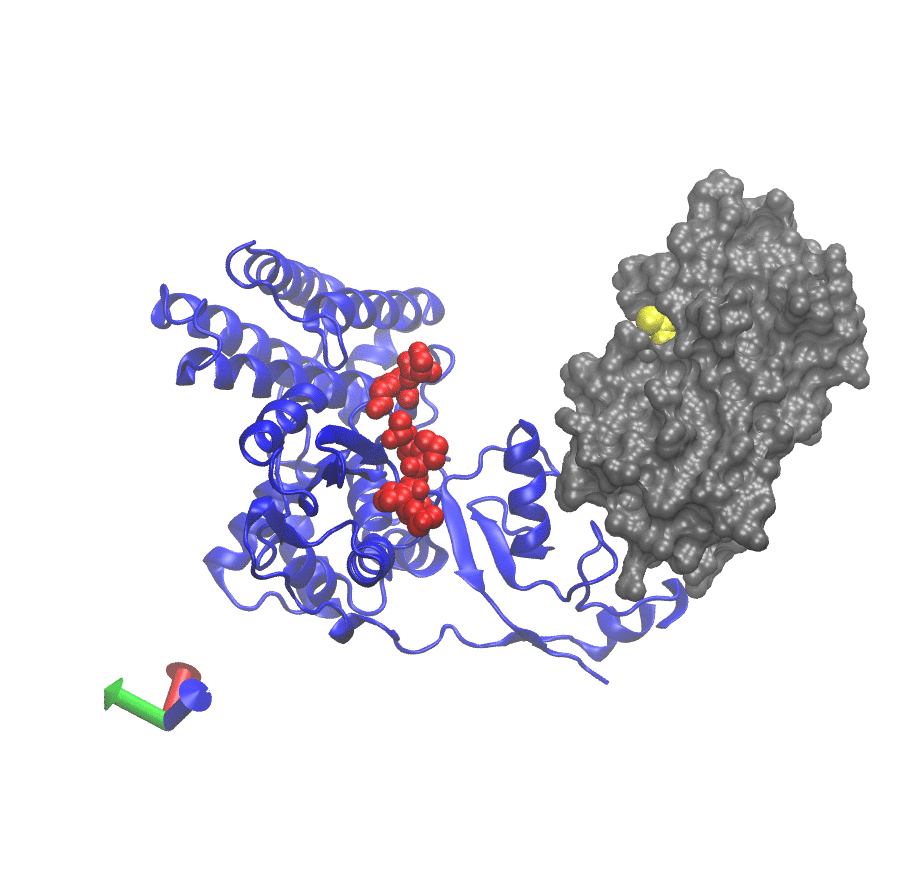

Supplement: Supplementary Zip Files S1 — Animated gif image files (labelled 4A4C_nm1.gif to 4A4C_nm5.gif) showing the normal mode transitions along the lowest frequency normal modes 1 to 5. In these movies, the E3 ligase (c-CBL) is in blue cartoon representation, the E2 in grey colored surface representation (with the catalytic CYS85 in yellow), and the substrate peptide in red VDW representation. (ZIP) [file pone.0065443.s013.zip › 4A4C_nm2.gif]

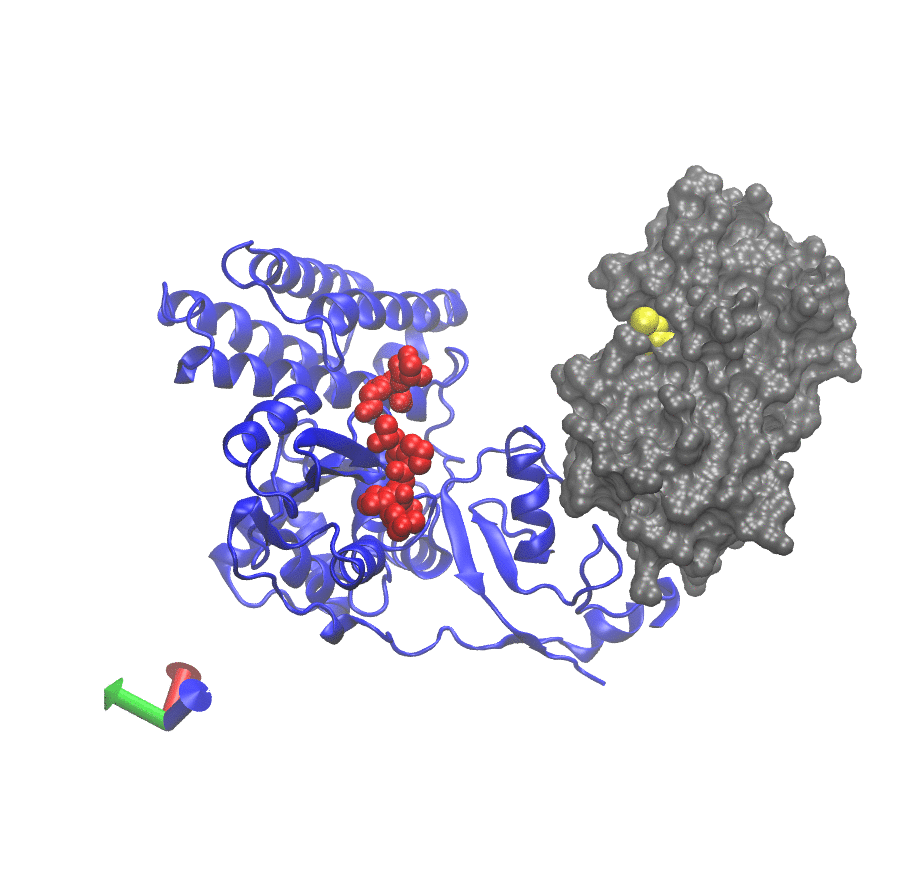

Supplement: Supplementary Zip Files S1 — Animated gif image files (labelled 4A4C_nm1.gif to 4A4C_nm5.gif) showing the normal mode transitions along the lowest frequency normal modes 1 to 5. In these movies, the E3 ligase (c-CBL) is in blue cartoon representation, the E2 in grey colored surface representation (with the catalytic CYS85 in yellow), and the substrate peptide in red VDW representation. (ZIP) [file pone.0065443.s013.zip › 4A4C_nm3.gif]

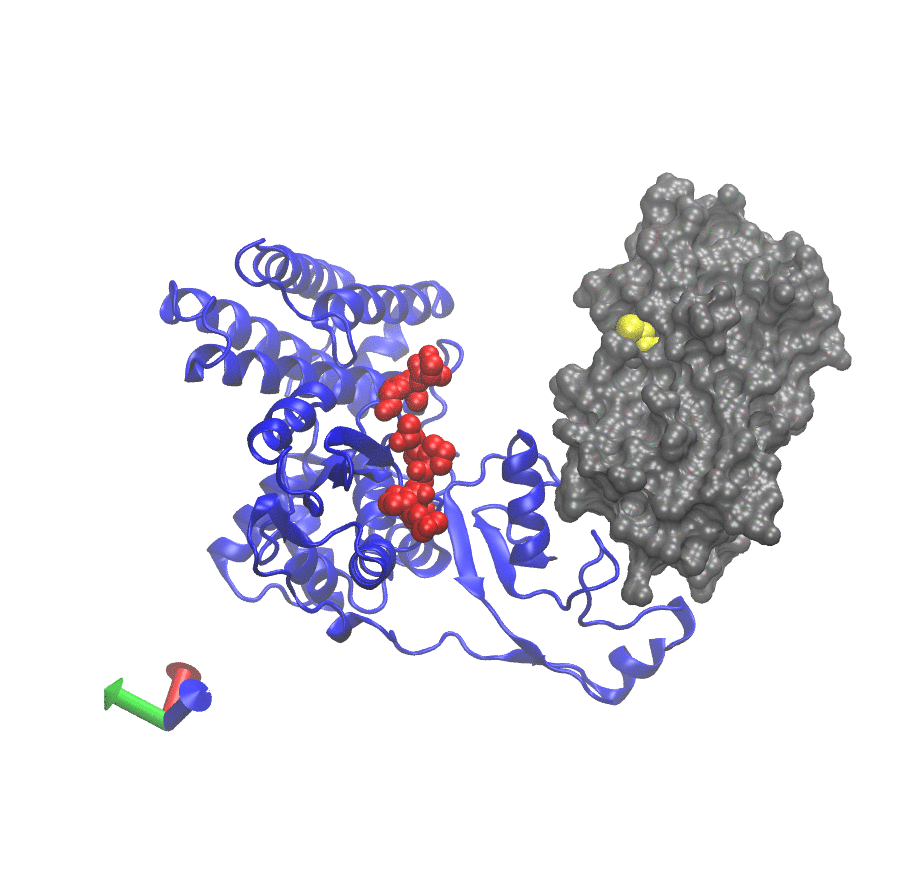

Supplement: Supplementary Zip Files S1 — Animated gif image files (labelled 4A4C_nm1.gif to 4A4C_nm5.gif) showing the normal mode transitions along the lowest frequency normal modes 1 to 5. In these movies, the E3 ligase (c-CBL) is in blue cartoon representation, the E2 in grey colored surface representation (with the catalytic CYS85 in yellow), and the substrate peptide in red VDW representation. (ZIP) [file pone.0065443.s013.zip › 4A4C_nm4.gif]

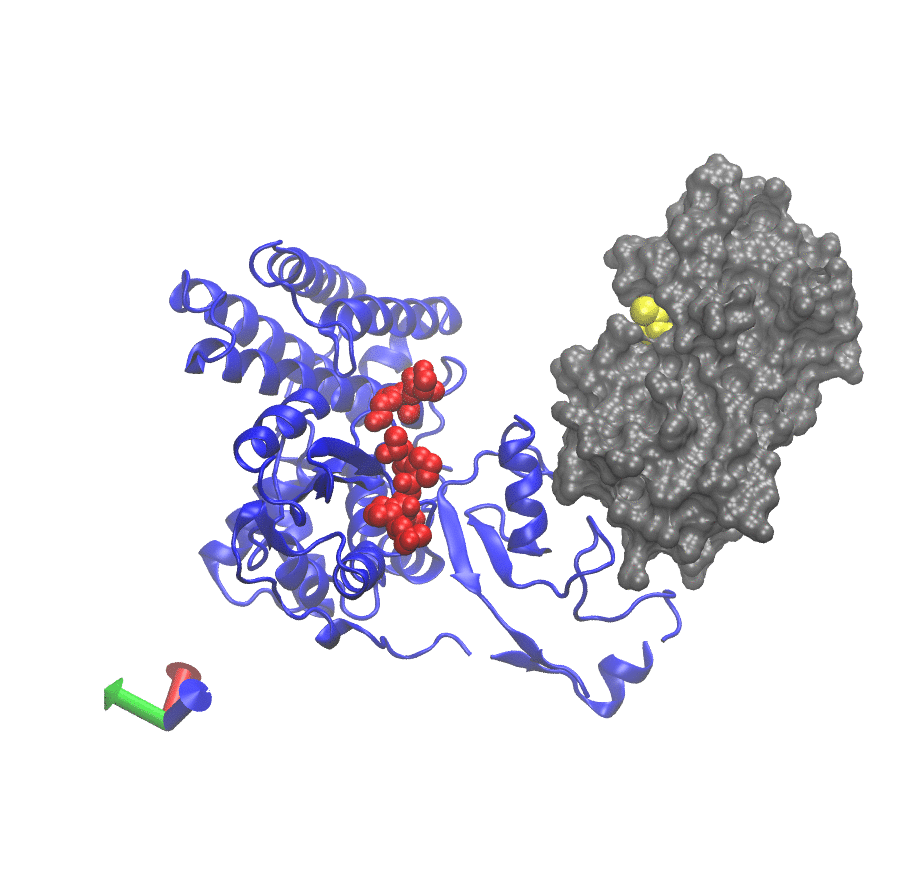

Supplement: Supplementary Zip Files S1 — Animated gif image files (labelled 4A4C_nm1.gif to 4A4C_nm5.gif) showing the normal mode transitions along the lowest frequency normal modes 1 to 5. In these movies, the E3 ligase (c-CBL) is in blue cartoon representation, the E2 in grey colored surface representation (with the catalytic CYS85 in yellow), and the substrate peptide in red VDW representation. (ZIP) [file pone.0065443.s013.zip › 4A4C_nm5.gif]
