# Supplementary material for: Functional Diversity and Structural Disorder in the Human Ubiquitination Pathway
Source: PLoS One. 2013 May 29;8(5):e65443. doi: 10.1371/journal.pone.0065443 (PMC3667038; doi:10.1371/journal.pone.0065443)
Supplement: Supplementary Zip Files S1 — Animated gif image files (labelled 4A4C_nm1.gif to 4A4C_nm5.gif) showing the normal mode transitions along the lowest frequency normal modes 1 to 5. In these movies, the E3 ligase (c-CBL) is in blue cartoon representation, the E2 in grey colored surface representation (with the catalytic CYS85 in yellow), and the substrate peptide in red VDW representation. (ZIP) [file pone.0065443.s013.zip › readme.rtf]

REAME: zip fileZip folder containing animated gif image files (labelled 4A4C_nm1.gif to 4A4C_nm5.gif) showing the normal mode transitions along the lowest frequency normal modes 1 to 5. In these movies, the E3 ligase (c-CBL) is in blue cartoon representation, the E2 in grey colored surface representation (with the catalytic CYS85 in yellow), and the substrate peptide in red VDW representation.
